# Ablation plus immunotherapy versus immunotherapy alone in patients of advanced NSCLC who develop oligo-residual disease after anti-PD-1/L1 therapy (BOOSTER): a randomized phase 2 trial

**DOI:** 10.1038/s41392-025-02460-z

**Published:** 2025-11-11

**Authors:** Shuo Yang, Xinyu Liu, Jia Yu, Xiaoxia Chen, Xiaozhen Liu, Sha Zhao, Tao Jiang, Hui Sun, Menghang Yang, Fengying Wu, Aiwu Li, Lei Wang, Guanghui Gao, Yaping Xu, Bin Chen, Shengxiang Ren

**Affiliations:** 1 Department of Medical Oncology, Shanghai Pulmonary Hospital, School of Medicine, Tongji University, Shanghai, China; 2 Department of Radiotherapy, Shanghai Pulmonary Hospital, School of Medicine, Tongji University, Shanghai, China

**Keywords:** Lung cancer, Cancer therapy

## Abstract

Local consolidative therapy (LCT) has been demonstrated to enhance the survival benefits of immunotherapy in non-small cell lung cancer (NSCLC) patients with oligometastatic or oligoprogressive disease. This randomized, phase 2 trial investigated the efficacy and safety of ablation combining continuous immunotherapy in NSCLC patients with oligo-residual disease (ORD) after anti-PD-1/L1 therapy (ChiCTR, identifier: ChiCTR2000032479). From March 2021 to March 2024, 65 patients were randomly assigned (2:1) to ablation combination group (*n* = 43) and immunotherapy maintenance group (*n* = 22), and the full analysis set finally included 42 patients in ablation plus immunotherapy group and 20 patients in immunotherapy maintenance group. With a median follow-up duration of 17.8 months, patients receiving ablation combination were associated with significantly longer PFS (median 26.7 vs. 11.7 months, *p* < 0.001, HR = 0.213, 95%CI 0.099–0.461) and a trend of longer OS (*p* = 0.036, HR = 0.242, 95%CI 0.057–1.019) than those without ablation. Subgroup analysis showed that cryoablation (*n* = 13) yielded potentially superior survival than thermal ablation (*n* = 31) (mPFS: NA vs. 22.4 months, *p* = 0.011), which might induced by the elevated level of IFN-α after cryotherapy compared to thermal ablation (*p* = 0.078). Additionally, ablation combination group showed a decreased rate of systemic progression pattern compared with immunotherapy maintenance group. Regarding safety, the combination of ablation and immunotherapy was well tolerated, with only 1 patient experiencing grade 3 pneumothorax after ablation. In conclusion, the addition of ablation is well-tolerated and prolongs the survival of immunotherapy in patients with advanced NSCLC who develop ORD after anti-PD-1/L1 therapy, while cryoablation showing potentially superior survival benefit compared to thermal ablation.

## Introduction

Currently, immunotherapy has become the backbone of lung cancer treatment.^1,2^ Immune checkpoint inhibitors (ICIs) targeting programmed death-1 (PD-1)/programmed death-ligand 1 (PD-L1), either as monotherapy or in combination with chemotherapy, have become the first-line standard of care for advanced non-small cell lung cancer (NSCLC) lacking driver gene alterations.^3,4^

However, resistance to immunotherapy inevitably develops in more than 80% of patients, and therapeutic options after resistance remain limited.^2,5^ The standard second-line chemotherapy yields a response rate of only about 10%, with a median progression-free survival (PFS) of less than 3 months.^6^ These challenges highlighted an urgent need to develop novel first-line immunotherapy combination strategies to further enhance therapeutic outcomes.

Notably, in recent years, local consolidative therapy (LCT) has emerged as a crucial approach in the era of immunotherapy. Several trials have demonstrated that radiotherapy, as a classical modality LCT, could significantly prolong PFS and overall survival (OS) in patients with oligo-progression of immunotherapy, which occurs in over 50% of cases.^7–11^ Both oligo-progression and oligo-residual belong to oligo-metastases. However, unlike oligo-progression, oligo-residual disease (ORD) refers to the small cluster of tumor cells that remain confined to limited sites after an initial treatment response. These lesions contains therapy-resistant subclones, which would serve as the potential seed for subsequent progression.^12^ Therefore, applying local therapy to ORD lesions may effectively eradicate resistant clones and enhance therapeutic efficacy. Indeed, it has been demonstrated that LCT for ORD could significantly prolong the survival in the era of targeted therapies. For instance, in EGFR-mutant lung cancer, the addition of LCT has been shown to extend the PFS of EGFR-TKIs treatment from 13.9 months to 20.6 months.^13–15^ However, the application of LCT for ORD in the setting of immunotherapy remains largely unexplored.

Besides radiotherapy, ablation has also been recognized as an important modality of LCT, with the advantages of being minimally invasive, providing a one-time solution, and causing relatively mild side effects.^16^ Depending on the energy modality, ablation could be categorized into cryoablation and thermal ablation (including radiofrequency, microwave, and laser ablation). These approaches utilize extreme temperatures to induce coagulative necrosis of tumor cells, thereby directly eradicating tumor lesions and reducing tumor burden. Importantly, ablation has also been shown to induce immunogenic cell death and trigger the release of tumor antigens, tumor necrosis factor-α and heat shock proteins, which act as “danger signals” to activate systemic anti-tumor immune responses.^16,17^ Furthermore, several reports including ours have documented a durable abscopal effect in a NSCLC patient treated with ablation after developing resistance to immunotherapy,^18,19^ highlighting the potential synergy between ablation and immunotherapy in patients with ORD.^20^

Randomized controlled trials investigating the clinical impact of ablation in patients with ORD have not been published yet. Considering the aforementioned success of ablative radiotherapy for oligo-progressive disease and the recent advances in elucidating the role of tumor heterogeneity in therapeutic resistance,^21–25^ we hypothesized that the addition of ablation to ORD may serve as a novel strategy to enhance the efficacy of immunotherapy in advanced NSCLC. In this study, we report the BOOSTER clinical trial—a randomized, phase II study to assess the efficacy and safety of combining ablation with ICIs for advanced NSCLC patients with ORD. As an exploratory objective, we also elucidated the patterns of disease progression and immunogenic changes after treatment.

## Results

### Patient enrollment and baseline characteristics

**Fig. 1:**
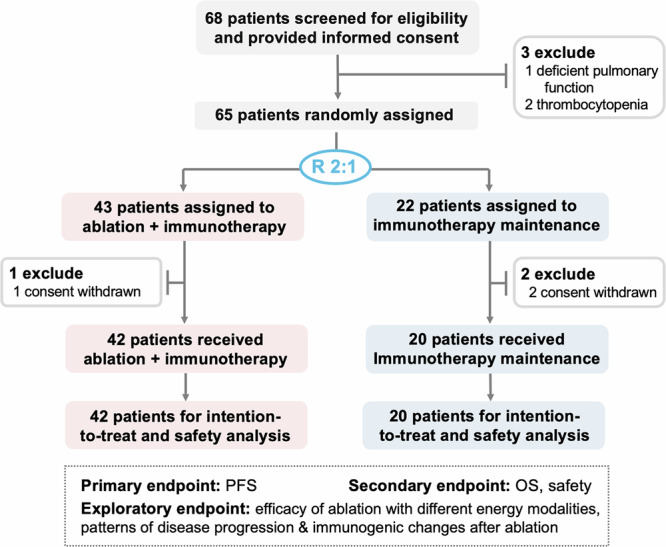
Trial profile. A total of 68 patients met the inclusion criteria, of whom 65 were randomized to either the ablation plus immunotherapy group (*n* = 43) or the immunotherapy maintenance group (*n* = 22). Ultimately, 42 patients in the ablation plus immunotherapy group and 20 in the immunotherapy group were included in the final analysis

From March 3rd 2021 to March 15th 2024, 152 out of 618 patients who received first-line immunotherapy and achieved ORD were further screened. Among them, 68 patients who met the inclusion criteria were provided informed consent and finally 65 patients were randomly assigned to either the ablation plus immunotherapy group (*n* = 43) or the immunotherapy maintenance group (*n* = 22). 1 patient in the ablation plus immunotherapy group and 2 patients in the immunotherapy maintenance group withdrew consent prior to initiating treatment and were therefore excluded. Ultimately, 42 patients in the ablation plus immunotherapy group and 20 patients in the immunotherapy group were included in the final analysis (Fig. 1).

The groups were well balanced for baseline characteristics (Table 1). Regarding the best response to immunotherapy, most patients achieved partial response (PR), with no significant difference between the two groups (80.9% vs. 75.0%, *p* = 0.741). Additionally, the number of immunotherapy cycles prior to randomization was comparable between the two groups (median: 6 vs. 6, *p* = 0.588) (Supplementary Fig. 1).

**Table 1:** Clinicopathological characteristics of patients enrolled

Characteristics	Total (*n*, %)	With ablation (*n*, %)	Without ablation (*n*, %)
Gender			
male	55 (88.7)	36 (85.7)	19 (95.0)
female	7 (11.3)	6 (14.3)	1 (5.0)
Age (years)			
<65	33 (53.2)	22 (52.4)	11 (55.0)
≥65	29 (46.8)	20 (47.6)	9 (45.0)
ECOG-PS			
0	20 (32.3)	14 (33.3)	6 (30.0)
1	42 (67.7)	28 (66.7)	14 (70.0)
Histology			
adenocarcinoma	38 (61.3)	25 (59.5)	13 (65.0)
squamous carcinoma	17 (27.4)	11 (26.2)	6 (30.0)
nsclc and other	7 (11.3)	6 (14.3)	1 (5.0)
Treatment			
IO + Chemo	59 (95.2)	39 (92.8)	20 (100.0)
IO mono	2 (3.2)	2 (4.8)	0 (0)
IO + Chemo + antiVEGF	1 (1.6)	1 (2.4)	0 (0)
PD-L1 expression			
unknown	18 (29.0)	12 (28.6)	6 (30.0)
negative	16 (25.8)	8 (19.0)	8 (40.0)
1–50%	15 (24.2)	12 (28.6)	3 (15.0)
≥50%	13 (21.0)	10 (23.8)	3 (15.0)
Oncogenic mutation			
unknown	18 (29.0)	12 (28.6)	6 (30.0)
wild type	29 (46.8)	19 (45.2)	10 (50.0)
KRAS	12 (19.4)	10 (23.8)	2 (10.0)
others^a^	3 (4.8)	1 (2.4)	2 (10.0)
Disease extent			
intrathoracic	34 (54.8)	26 (61.9)	8 (40.0)
extrathoracic	28 (45.2)	16 (38.1)	12 (60.0)
ICIs			
Camrelizumab	44 (71.0)	32 (76.2)	12 (60.0)
Tislelizumab	10 (16.1)	5 (11.9)	5 (25.0)
Pembrolizumab	8 (12.9)	5 (11.9)	5 (25.0)
Total	62	42	20

*ECOG* Eastern Cooperative Oncology Group, *PS* performance status, *ICIs* immune checkpoint inhibitors

^a^one with HER2 mutation, one with MET amplification and one with RET fusion

### Efficacy

**Fig. 2:**
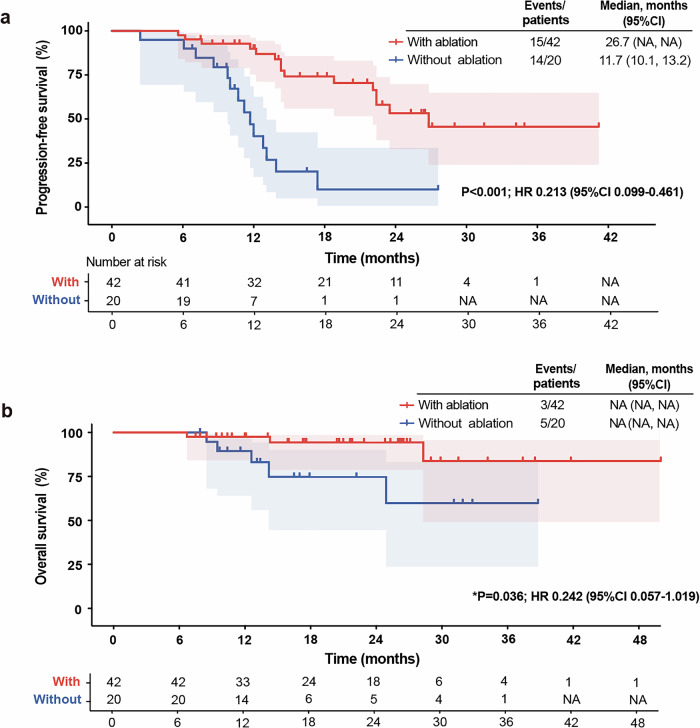
Efficacy of study. **a** Kaplan–Meier curve of PFS for ablation group (*n* = 42) and non-ablation group (*n* = 20) since immunotherapy initiation. **b** Kaplan–Meier curve of OS for ablation group (*n* = 42) and non-ablation group (*n* = 20) since immunotherapy initiation. Schaded areas represent 95% CIs. HR hazard ratio, NA not available, CI confidence interval. *P refers to nominal *P* value

The median duration of follow-up was 17.8 months. The median PFS of immunotherapy was 26.7 months (95% CI NA) in the ablation group versus 11.7 months (95% CI 10.1–13.2) in the non-ablation group (*p* < 0.001, HR = 0.213, 95%CI 0.099–0.461). Although OS was not reached, patients who received ablation showed a trend of prolonged OS than those without ablation (NA vs. NA, *p* = 0.036, HR = 0.242, 95% CI 0.057–1.019) (Fig. 2). Furthermore, to account for the potential impact of the number of immunotherapy cycles prior to randomization, we also evaluated PFS and OS since randomization, both of which were superior in the ablation group (mPFS: 17.1 vs. 4.4, *p* < 0.001, HR = 0.198, 95%CI 0.090–0.432; mOS: NA vs. NA, *p* = 0.032, HR = 0.234, 95% CI 0.055–0.993, Supplementary Fig. 2).

Additionally, although 3 patients who withdrew consent were not included in the primary analyses for not receiving the assigned intervention, we presented their immunotherapy cycles and time prior to randomization (Supplementary Table 3) and conducted supplementary survival analyses including all 65 randomized patients (Supplementary Figs. 3, 4).

The results of the power analysis indicated that with the first 36 out of 137 PFS events, a current *Z* value of 4.26 would achieve 99.51% predictive power of success.

### Subgroup analysis of survival benefits

**Fig. 3:**
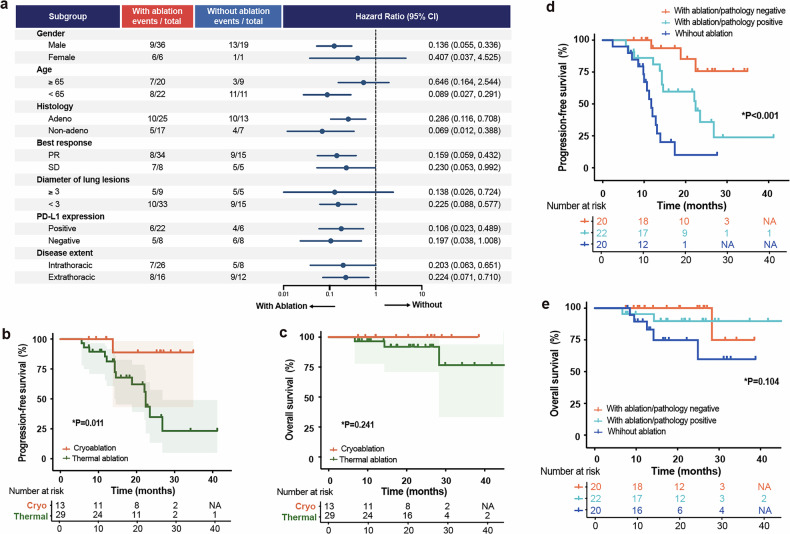
Subgroup analysis. **a** Forest plot of the subgroup analysis by baseline characteristics for progression-free survival. **b**, **c** Survival analysis comparing PFS and OS in patients with cryoablation (*n* = 13) versus thermal ablation (*n* = 29). **d**, **e** Survival analysis comparing PFS and OS in patients ablation/pathology negative (*n* = 20) versus with ablation/pathology positive (*n* = 22) versus without ablation (*n* = 20). Shaded areas represent 95% CIs. NA not available, CI confidence interval, Cryo cryoablation, Thermal thermal ablation. *P refers to nominal *P* value

The subgroup analysis revealed that nearly all of the major subgroups tended to favored for the combination of ablation and immunotherapy (Fig. 3a). Besides, the survival analysis showed that cryoablation was associated with a trend of longer PFS (mPFS: NA vs. 22.4 months, *p* = 0.011) and improved OS (mOS: NA vs.NA, *p* = 0.241) compared to thermal ablation (Fig. 3b, c, Supplementary Tables 1, 2). Additionally, all patients in the ablation group underwent pre-ablation biopsy. Patients with negative pathology showed a trend toward prolonged PFS (median: NA vs. 22.4 vs. 11.7 months, *p* < 0.001) and OS (mOS: NA vs.NA, *p* = 0.104) compared to those with positive pathology or no ablation (Fig. 3d, e). Additionally, two patients with negative pathology underwent PET-CT before ablation, both of whom demonstrated low FDG uptake, with SUVmax values of 1.64 and 2.08, respectively (Supplementary Fig. 5).

### Patterns of disease progression

**Fig. 4:**
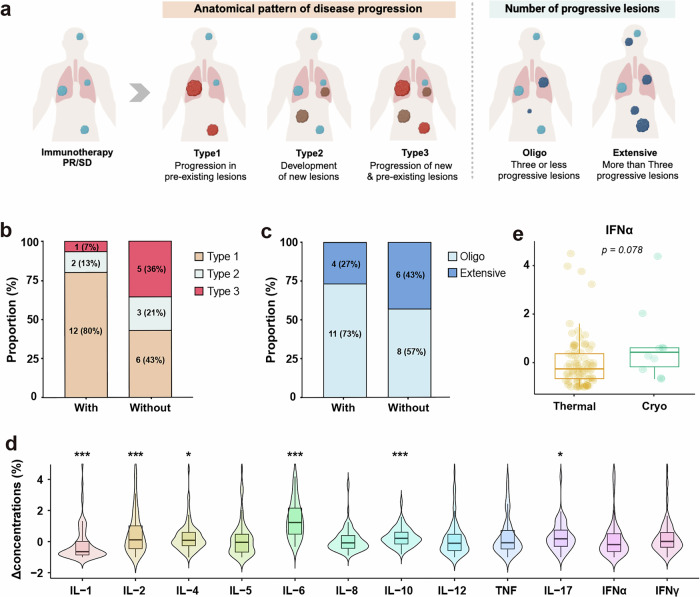
Patterns of disease progression and immunogenic changes after ablation. **a** Illustration of distinct patterns of disease progression. **b** Anatomical pattern of disease progression of with ablation group and without ablation group. **c** Pattern of disease progression based on the number of progressive lesions in with ablation group and without ablation group. **d** Changes in cytokine levels pre and post ablation. **e** Changes in IFN-α levels pre and post thermal ablation and cryoablation. The Δconcentration(%) is calculated with a formula of (cytokine level post ablation-cytokine level pre ablation)/cytokine level pre ablation. Statistical analysis was performed using Student’s *t*-test. * represents *p* < 0.05, ** represents *p* < 0.01, *** represents *p* < 0.001

The addition of ablation to immunotherapy may alter the patterns of disease progression. First, we assessed the anatomical progression pattern, categorizing it into three types based on the location of the progressed lesions: Type 1, progression in pre-existing lesions; Type 2, development of new lesions; and Type 3, progression in both pre-existing and new lesions (Fig. 4a). Notably, patients who received ablation showed a reduced proportion of Type 3 progression (7% vs. 36%) and an increased proportion of Type 1 progression (80% vs. 43%, Fig. 4b). Furthermore, when evaluating progression as oligo- or extensive based on the number of progressed lesions, ablation was associated with a reduced proportion of extensive progression (27% vs. 43%, Fig. 4c). Of note, progression in ablation-treated lesions was observed in 26.7% (4/15) of patients.

### Immunogenic changes after ablation

To better characterize the immunogenic changes induced by ablation, we collected pre- and post-ablation peripheral blood samples from a cohort of 97 patients with advanced NSCLC (Supplementary Table 4). The levels of IL-2, IL-4, IL-6, IL-10 and IL-17 were significantly increased after ablation, whereas the IL-1β level was decreased (Fig. 4d). The cytokine changes were also analyzed based on the different ablation modalities (Supplementary Fig. 6a, b). Given the potential survival benefit of cryoablation over thermal ablation, we further compared the percentage changes in cytokine levels between the two modalities, which revealed that IFN-α was the only cytokine that showed a trend of elevated level in the cryoablation group (Fig. 4e, Supplementary Fig. 4c).

### Safety

The most common immunotherapy-related adverse events were fatigue (11/62, 17.7%), decreased appetite (9/62, 14.5%), asthenia (9/62, 14.5%), pneumonia (8/62, 12.9%) and anemia (8/62, 12.9%) in both groups. Immunotherapy-related adverse events of grade 3 or higher in both groups were fatigue (2.3%, 5.0%), pneumonia (2.3%, 5.0%). And anemia (1/42, 2.3%), nausea (1/42, 2.3%) and constipation (1/42, 2.3%) were only reported in the ablation group. As for ablation-related adverse events, pneumothorax (15/42, 35.7%), pleural effusion (10/42, 23.8%), bronchopulmonary hemorrhage (8/42, 19.0%), pleurodynia (6/42, 14.3%), asthenia (6/42, 14.3%), decreased appetite (5/42, 11.9%), fatigue (4/42, 9.5%), pneumonia (2/42, 4.8%) were reported. Notably, 1 patient was observed with grade 3 pneumothorax (Table 2).

**Table 2:** Summary of adverse events

	With ablation (*n* = 42)		Without ablation (*n* = 20)	
	All grades	Grade ≥ 3	All grades	Grade ≥ 3
**AEs related to immunotherapy**				
Fatigue	8 (19.0)	1 (2.3)	3 (15.0)	1 (5.0)
Asthenia	6 (14.3)	0	3 (15.0)	0
Decreased appetite	6 (14.3)	0	3 (15.0)	0
Pneumonia	6 (14.3)	1 (2.3)	2 (10.0)	1 (5.0)
Anemia	5 (11.9)	1 (2.3)	3 (15.0)	0
Nausea	5 (11.9)	1 (2.3)	2 (10.0)	0
Constipation	4 (9.5)	1 (2.3)	2 (10.0)	0
Rash	4 (9.5)	0	2 (10.0)	0
Thrombocytopenia	4 (9.5)	0	1 (5.0)	0
Neutropenia	3 (7.1)	0	1 (5.0)	0
Hypothyroidism	3 (7.1)	0	1 (5.0)	0
**AEs related to ablation**				
Pneumothorax	15 (35.7)	1 (2.3)		
Pleural effusion	10 (23.8)	0		
Pleurodynia	8 (19.0)	0		
Bronchopulmonary hemorrhage	6 (14.3)	0		
Asthenia	6(14.3)	2 (4.8)		
Decreased appetite	5 (11.9)	2 (4.8)		
Fatigue	4 (9.5)	1 (2.3)		
Pneumonia	2 (4.8)	0		

## Discussion

As far as we know, the BOOSTER study is the first randomized phase II trial to evaluate the efficacy and safety of ablation plus continuous immunotherapy in advanced NSCLC patients who developed ORD after anti-PD-1/L1 therapy. We found that ablation significantly prolonged the progression free survival (26.7 vs 11.7 months) in this population, with cryoablation offering a trend toward superior benefits compared to thermal ablation. Ablation appeared to decrease systemic progression and IFN-α was potentially associated with better efficacy of cryoablation. Meanwhile, the addition of ablation was generally well tolerated, with no unexpected adverse events.

ORD after treatment may serve as the source of clonal evolution and the origin of disease progression following immunotherapy, representing a critical clinical challenge. In this study, we found that 24.6% of advanced NSCLC patients developed ORD after immunotherapy, which is consistent with previous reports.^12^ Importantly, we found that applying ablation to ORD doubled median PFS two times compared to immunotherapy maintenance alone (26·7 vs 11·7 months). Furthermore, the two groups had distinct patterns of disease progression. Specifically, local progression was more common in the ablation group, whereas systemic progression predominated in the observation group, highlighting the potential role of local therapy in overcoming tumor evolution. Besides, the progression pattern in ablation group provided patients with greater opportunities for re-administration of LCT, potentially offering survival advantages over switching systemic treatment strategies.^26^

However, the recent NRG002Lung study showed that radiotherapy and surgical resection, two common approaches to LCT, failed to enhance the efficacy of immunotherapy.^27^ The negative results of NRG002Lung study revealed the complexity of managing ORD in the context of immunotherapy and underlined the need for further research to optimize LCT strategies in this setting. These controversial results may be attributed to differences in patient cohorts and study designs. (1) Regarding patient cohorts, only 33.1% of patients in the NRG002Lung trial achieved a PR to first-line systemic therapy, compared to 89% in our study. Besides, 47.6% of patients in our study were with negative pathology when receiving ablation. Our subgroup analysis indicated that patients with PR and negative pathology were more likely to benefit from the combination strategy. Thus, the highly selected patients after immunotherapy would be benefited from local ablation therapy. (2) In terms of study design, NRG002Lung study applied radiotherapy to all tumor sites, while our study only administrered ablation to limited lung lesions. Treating all tumor sites likely increased adverse events and impaired systemic antitumor immunity due to T-cell depletion in locally draining lymph nodes. The pooled analysis of PEMBRO-RT and MDACC trials showed that radiotherapy to limited tumor sites improved PFS and OS, with a 41.7% abscopal response rate. This finding also suggested, to some extent, that localized LCT may better enhance long-term immune responses.^28^

Both thermal ablation and cryoablation were adopted in this study, with 13 patients receiving cryoablation and 29 patients receiving thermal ablation, which included 6 with MWA and 23 with RFA. Subgroup analysis showed that patients were more likely to benefit from cryoablation (median PFS: NA vs. 22.4 months, *p* = 0.011). However, since no multiple comparison correction was applied and patients in the thermal ablation group had significantly larger lung lesions in our cohort (*p* = 0.033), the observed superiority of cryoablation requires further validation in a larger population. Accordingly, our ongoing phase II clinical trial (ChiCTR2500103025) is investigating the optimal ablation energy modality for lung cancer. Additionally, the immunogenic changes analysis indicated that the elevated levels of IFN-α after ablation may be the potential mechanism for the superior efficacy of cryoablation. A previous study also indicated that IFN-α and IFN-β enhanced the efficacy of immunotherapy by triggering type I interferon-dependent antitumor immunity in patients receiving cryoablation,^29^ which was consistent with our clinical findings.

Besides, in the ablation group, we also found that the patients with negative pathological findings had a trend of superior survival outcomes, and those with positive pathological findings also showed a survival benefit compared with the control group. As is well known, patients with negative pathological findings might have a pathological complete response (pCR). In the area of neoadjuvant immunotherapy, pCR is a key indicator of long-term survival, and CheckMate-816 study has also included pCR as one of its primary endpoints.^30^ Moreover, two patients who underwent PET-CT before ablation, both with negative pathology, showed SUVmax values of 1.64 and 2.08 respectively. These results suggest that in the context of ORD, PET-CT findings are consistent with pathology, as seen in neoadjuvant therapy.^31^ Nevertheless, given the limited number of patients in our cohort, particularly that only two underwent PET-CT, the significance of negative pathology in ORD requires further investigation.

Our study has several limitations. Firstly, despite our focus on patients with oligo-residual NSCLC and EGFR/ALK wildtype, there remains inherent heterogeneity, such as driver gene alteration status, histology, overall disease burden, metastatic disease interval, and previous systemic therapy. The finding in this study might not be generalizable to the whole population. Additionally, the sample size in our study was smaller than initially planned, which may have reduced statistical power. Nevertheless, post hoc power analysis indicated that the study retained over 99% predictive power, which supports the validity of the findings despite the reduced sample size. Lastly, our biomarker investigation collected paired blood sample at baseline and after ablation and evaluated the dynamic change of cytokines. As we know, the immune cells are more closed related with the effect and warrant further investigation in the future.

In summary, this BOOSTER study showed that the addition of LCT, thermal ablation or cryoablation, is well tolerated and leads to a clinically meaningful prolongation of PFS and OS compared with continuous immunotherapy alone in advanced NSCLC patients who develop ORD after anti-PD-1/PD-L1 therapy. To validate the efficacy of ablation in this setting, further research in a well-defined patient population is required through a phase III clinical trial.

## Materials and methods

### Study design and patients

This open-label, single center randomized phase II trial investigated the efficacy and safety of ablation plus immunotherapy versus immunotherapy in advanced NSCLC patients who developed ORD after anti-PD-1/PD-L1 therapy. All the participants were enrolled from Shanghai Pulmonary Hospital, Tongji University, Shanghai, China. The main eligibility criteria included: (1) age ≥18 years; (2) Eastern Cooperative Oncology Group (ECOG) performance score (PS) of 0 or 1; (3) histopathologically confirmed advanced/metastatic NSCLC (stage IIIB with multiple lobes involved/stage IV); (4) administrated with first-line immunotherapy monotherapy or in combination with chemotherapy; (5) achieved oligo-residual state after first-line therapy, defined as partial response (PR) or stable disease (SD) as the best response to immunotherapy, with residual tumors confined to a maximum of three organs and five lesions; (6) ORDs in lung were suitable for ablation; (7) Patients had no disease progression before randomization. Patients with symptomatic brain metastases, severe deficient pulmonary function and other serious/persistent comorbidities were excluded. Further inclusion and exclusion criteria were provided in the appendix.

The study was approved by the Ethics Committee of Shanghai Pulmonary Hospital (L20-244), and all participants were provided written approved consent before enrollment.

### Randomization and masking

Eligible patients were randomly assigned (2:1) into two groups: in the first group, participants received maintenance immunotherapy combined with ablation to the ORD, as determined by physician; in the second group, participants received maintenance immunotherapy alone. Randomization was stratified by age (<65 vs. ≥65) and ECOG PS (0 vs. 1). Neither patients nor investigators were masked to treatment assignments. However, the data analysts remained blinded to the treatment allocation until completion of preliminary analysis.

### Procedures

The best response of first-line immunotherapy (any immune checkpoint inhibitors recommended by the NCCN guideline, either as monotherapy or in combination with chemotherapy were eligible for use) was evaluated on the basis of Response Evaluation Criteria in Solid Tumors (RECIST) version 1.1. Patients were defined as having ORD based on the following criteria: (1) achieved the best overall response of partial response (PR) or stable disease (SD); (2) residual tumors were stable at the next evaluation after achieving the best response to immunotherapy, irrespective of the number of treatment cycles; (3) residual tumors confined to a maximum of three organs and five lesions. All ORDs in the lung that were eligible for ablation—assessed by a multidisciplinary tumor board including an ablation oncologist—were treated with ablation. However, ORDs in other sites did not undergo ablation. Immunotherapy was temporarily discontinued during ablation, and treatment was resumed with the original immunotherapy regimen once post-ablation inflammatory changes resolved, as confirmed by follow-up CT imaging. Immunotherapy was administrated until disease progression, intolerable toxicity, patient death, or consent withdrawal.

Both thermal ablation (including microwave and radiofrequency ablation) and cryoablation were permissible. The energy modalities, power and duration of ablation were determined by the ablation oncologist, based on the tumor size and location. For microwave ablation, the power was typically 50–60 W, with a duration of 6–10 min. For radiofrequency ablation, the procedure started at a lower power (20–30 W), gradually increasing until the peak impedance was reached. For cryoablation, at least two cycles were performed, with freezing times of 10–15 min and rewarming times of 3–5 min. Following the ablation procedure, a follow-up chest CT scan was conducted to assess whether the ablation area was fully covered, ensuring complete tumor ablation.

Peripheral blood samples of patients who received ablation at baseline and post-ablation (within 48 h) were collected and further tested for interleukin levels using flow cytometry via BD Cytometric Bead Array in the Clinical Laboratory Department of Shanghai Pulmonary Hospital.

### Outcomes

The primary endpoint was PFS, defined as the time from immunotherapy initiation to Response Evaluation Criteria in Solid Tumors-defined progression or death. Secondary endpoints included OS (the time from immunotherapy initiation to death from any causes or last follow-up) and safety (monitoring adverse events and laboratory abnormalities, adverse events were classified according to CTCAE 5.0 and ablation-related events were classified according to The Society of Interventional Radiology AE Classification System). As exploratory objectives, efficacy of ablation with different energy modalities (thermal ablation vs. cryoablation), patterns of disease progression (ablation to ORD vs. without ablation) and immunogenic changes after ablation were assessed.

### Statistical analysis

The sample size calculation was based on the assumption that the ablation plus immunotherapy would improve the median PFS of immunotherapy from 6 to 12 months, resulting in a hazard ratio (HR) of 0.500. Using 2-sample log-rank test, with randomization ratio as 2:1 and anticipated dropout rate of 10%, it was determined that a total sample size of 137 patients (91 patients for ablation plus immunotherapy and 46 for immunotherapy) would be needed to achieve 80% statistical power, with a two-sided of α 0.05, at which point a *p* value less than 0.05 would be considered to indicate a statistically significant difference between ablation plus immunotherapy group and immunotherapy group. Nevertheless, due to the slow recruitment and subsequent approval of Ivonescimab, which is expected to further advance immunotherapy, the trial ultimately recruited only 65 patients. Post hoc power analyses were conducted to assess the ability to detect an HR of 0.500 with an alpha level of 0.05 using a two-sided log-rank test, including the assessment of post hoc predictive power of success.

Comparisons of baseline characteristics used Chi-square or Fisher’s exact tests. Kaplan–Meier estimates were applied for median PFS/OS, with log-rank tests for between-group differences. Cox proportional hazards models provided hazard ratios (HRs) and 95% confidence intervals (CIs) for subgroup analyses. Changes in interleukin levels were assessed by Student’s *t*-test or Wilcoxon rank-sum test. A two-sided *p* < 0.05 was considered statistically significant. Analyses were performed using SPSS version 22.0 (SPSS Inc., Chicago, IL).

## Supplementary information

Supplementary figures & tables

Study protocol

## Data Availability

The data supporting the findings of this study could be available for scientific purpose from the corresponding author (harry_ren@tongji.edu.cn).
