# Supplementary material for: Ablation plus immunotherapy versus immunotherapy alone in patients of advanced NSCLC who develop oligo-residual disease after anti-PD-1/L1 therapy (BOOSTER): a randomized phase 2 trial
Source: Signal Transduct Target Ther. 2025 Nov 11;10:365. doi: 10.1038/s41392-025-02460-z (PMC12603194; doi:10.1038/s41392-025-02460-z)
Supplement: Supplementary file 1 — Supplementary figures & tables [file 41392_2025_2460_MOESM1_ESM.docx]

Supplementary Materials for

Ablation plus immunotherapy versus immunotherapy alone in patients of advanced NSCLC who develop oligo-residual disease after anti-PD-1/L1 therapy (Booster): a randomized phase 2 trial

Shuo Yang, Xinyu Liu, Jia Yu, Xiaoxia Chen, Xiaozhen Liu, Sha Zhao, Tao Jiang, Hui Sun, Menghang Yang, Fengying Wu, Aiwu Li, Lei Wang, Guanghui Gao, Yaping Xu, Bin Chen, Shengxiang Ren

Correspondence to: Shengxiang Ren, [harry_ren@tongji.edu.cn](mailto:harry_ren@tongji.edu.cn); Bin Chen, [binchen629@126.com](mailto:binchen629@126.com); Yaping Xu, [xuyaping1207@126.com](mailto:xuyaping1207@126.com);

**This PDF file includes:**

Materials

Figures. S1 to S6

Tables S1 to S4

Figure. S1. Baseline characteristics of patients. (a) Best response of immunotherapy prior randomization. (b) Cycles of immunotherapy prior randomization.
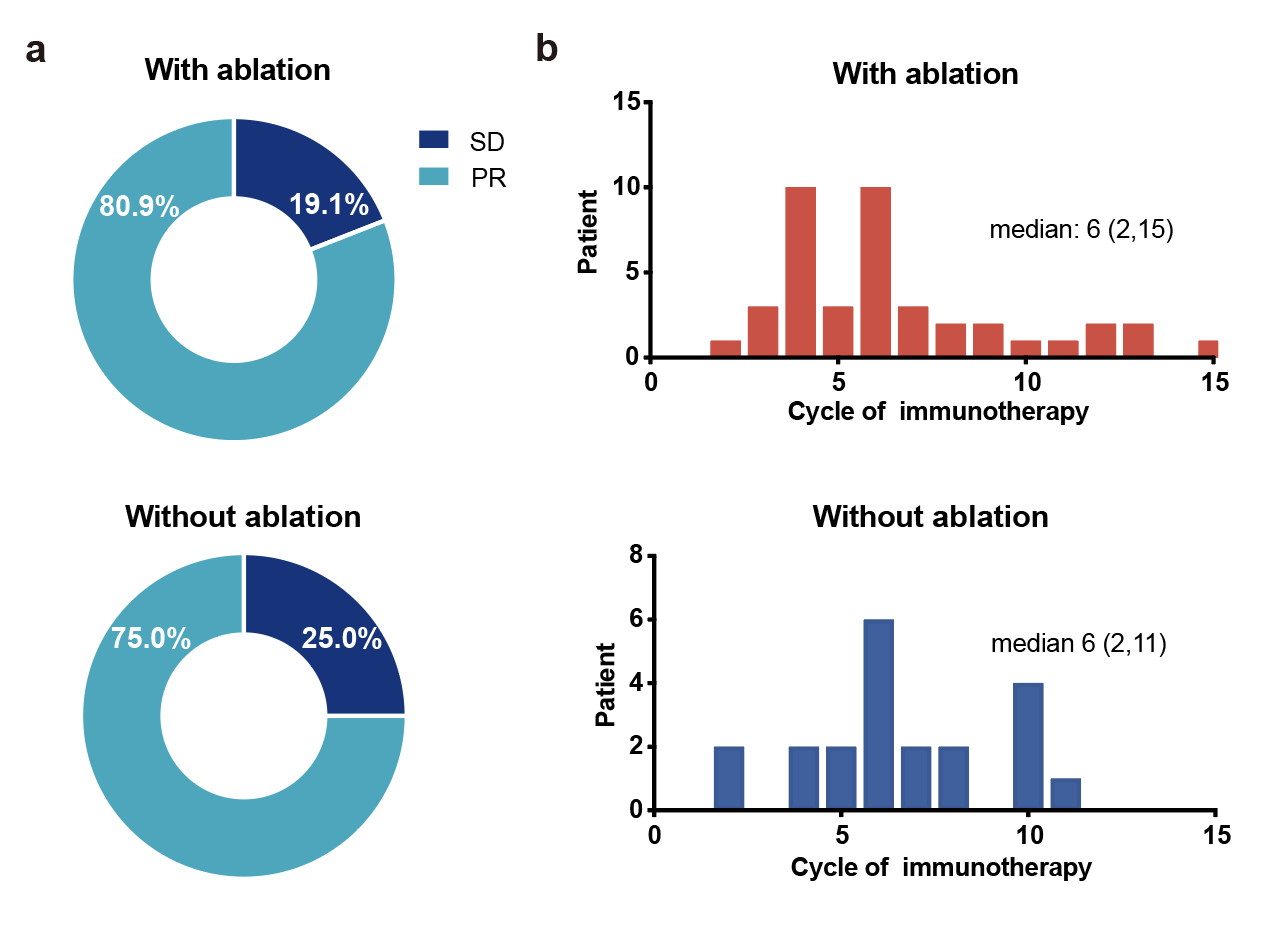


**Figure. S2.** Kaplan-Meier analysis since randomization. (a) Progression-free survival. (b) Overall survival. Shaded areas represent 95% CIs. HR: hazard ratio; NA: not available; CI: confidence interval. *P refers to nominal P value.

**
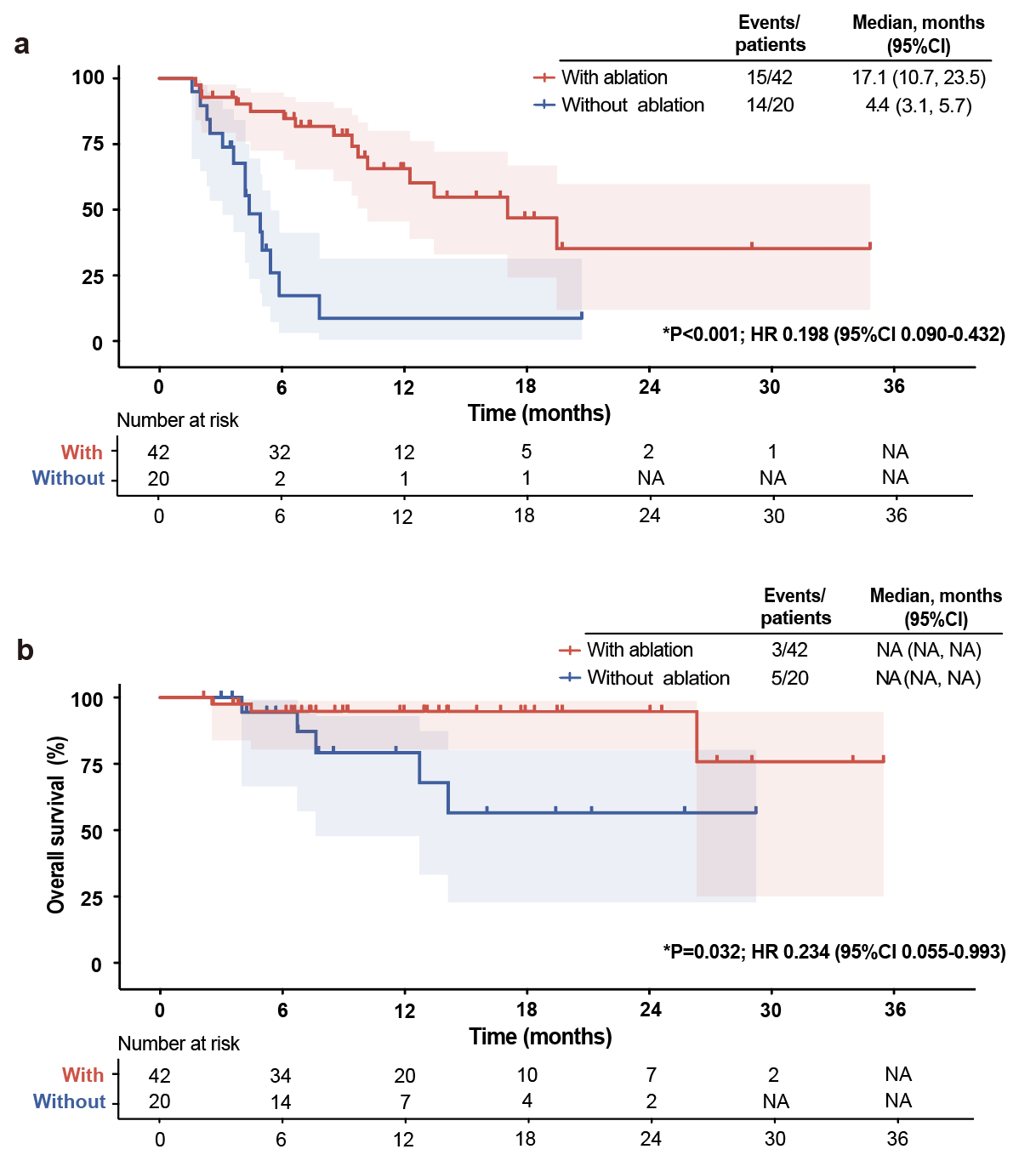
**

**Figure. S3.** Kaplan-Meier analysis since immunotherapy initiation in the 65 patients’ cohort. (a) Progression-free survival. (b) Overall survival. Shaded areas represent 95% CIs. HR: hazard ratio; NA: not available; CI: confidence interval. *P refers to nominal P value.


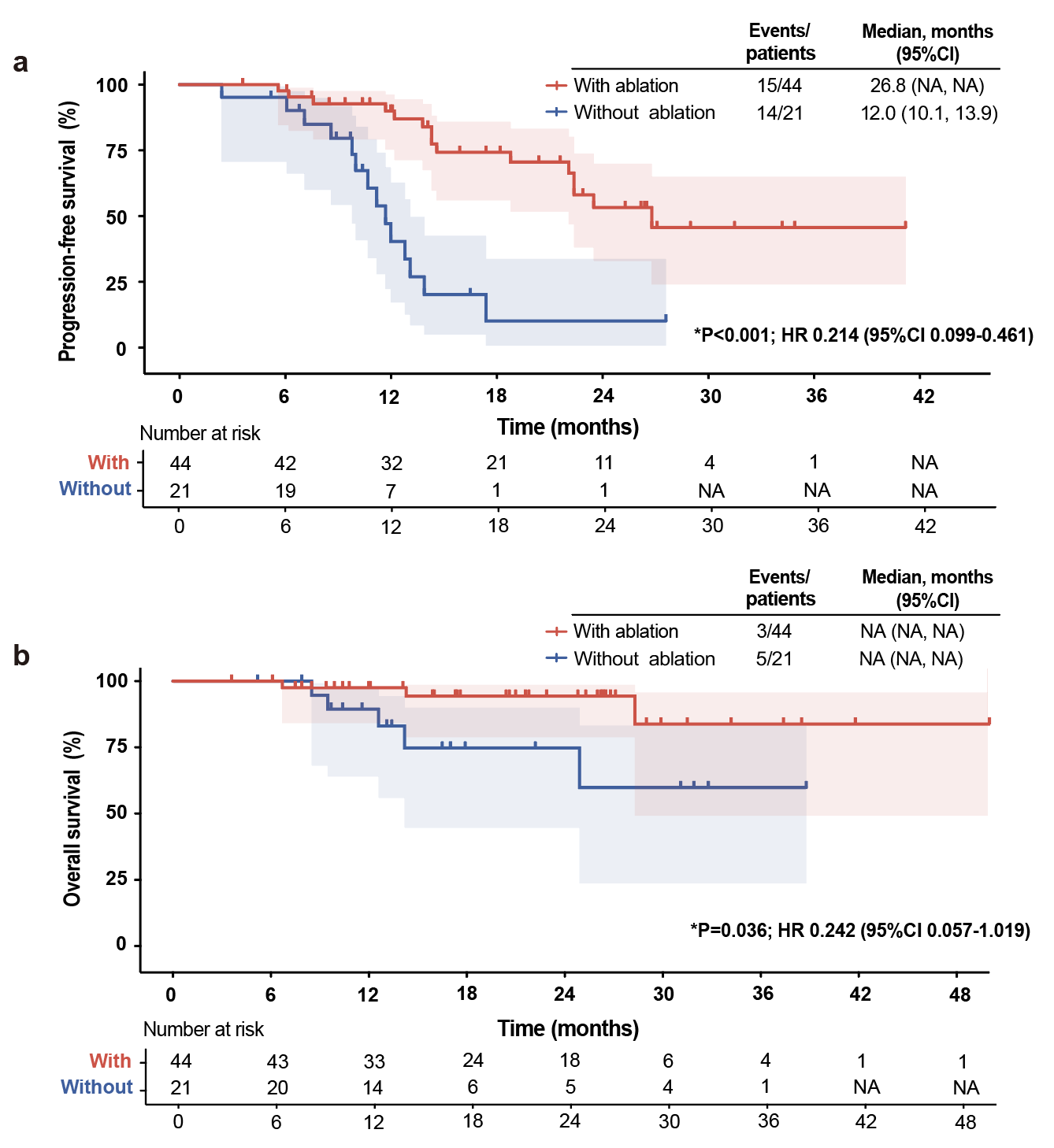


**Figure. S4.** Kaplan-Meier analysis since randomization in the 65 patients’ cohort. (a) Progression-free survival. (b) Overall survival. Shaded areas represent 95% CIs. HR: hazard ratio; NA: not available; CI: confidence interval. *P refers to nominal P value.


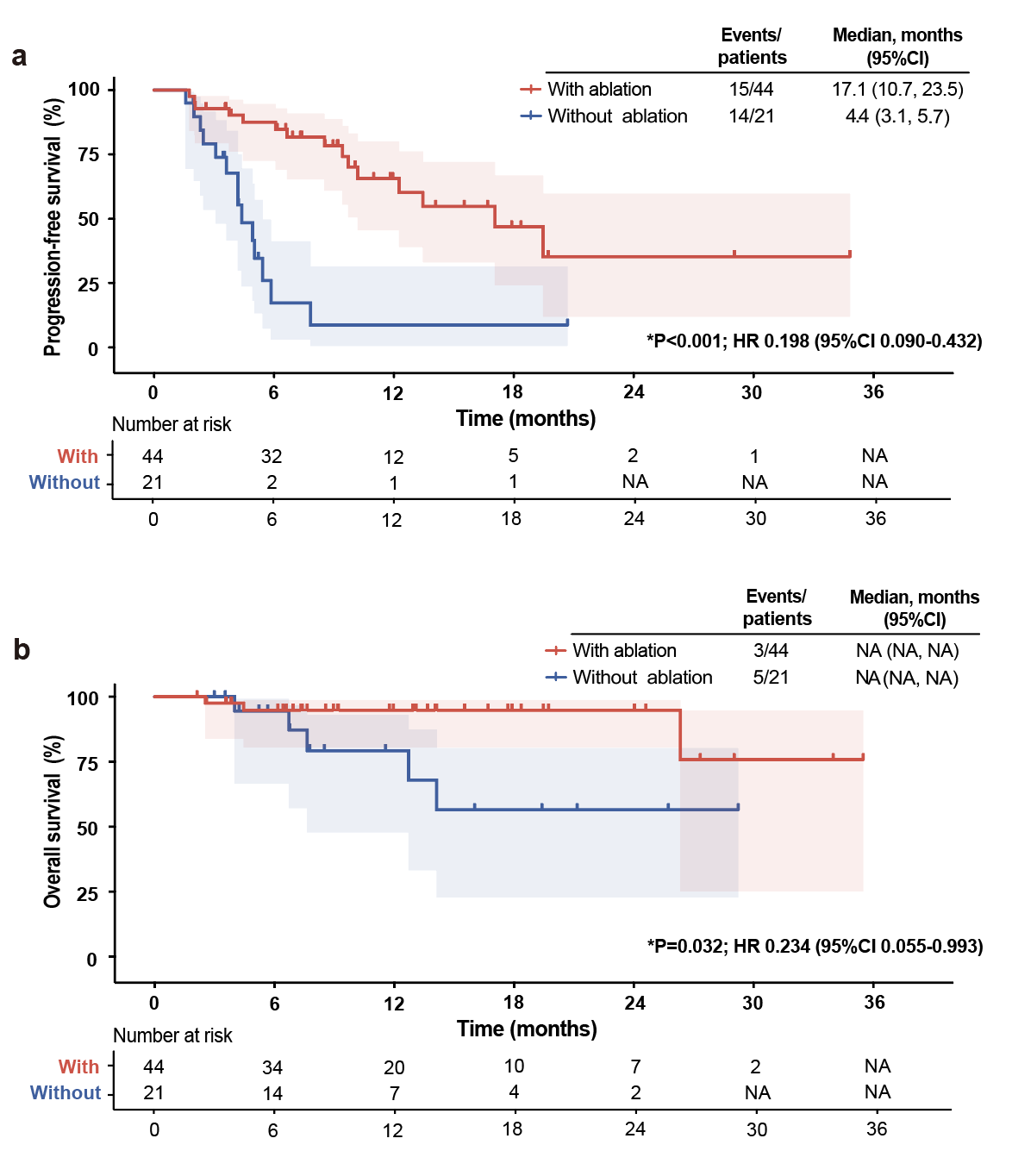


**Figure. S5.** The PET-CT images obtained before ablation from two patients with negative pathology. (a) A 64-year-old male with non-small cell lung cancer (NSCLC) who achieved a partial response (PR) following treatment with carrilizumab plus AC regimen, PET-CT images before ablation. (b) An 81-year-old male with lung squamous cell carcinoma who achieved a partial response (PR) after carrilizumab monotherapy, PET-CT images before ablation.


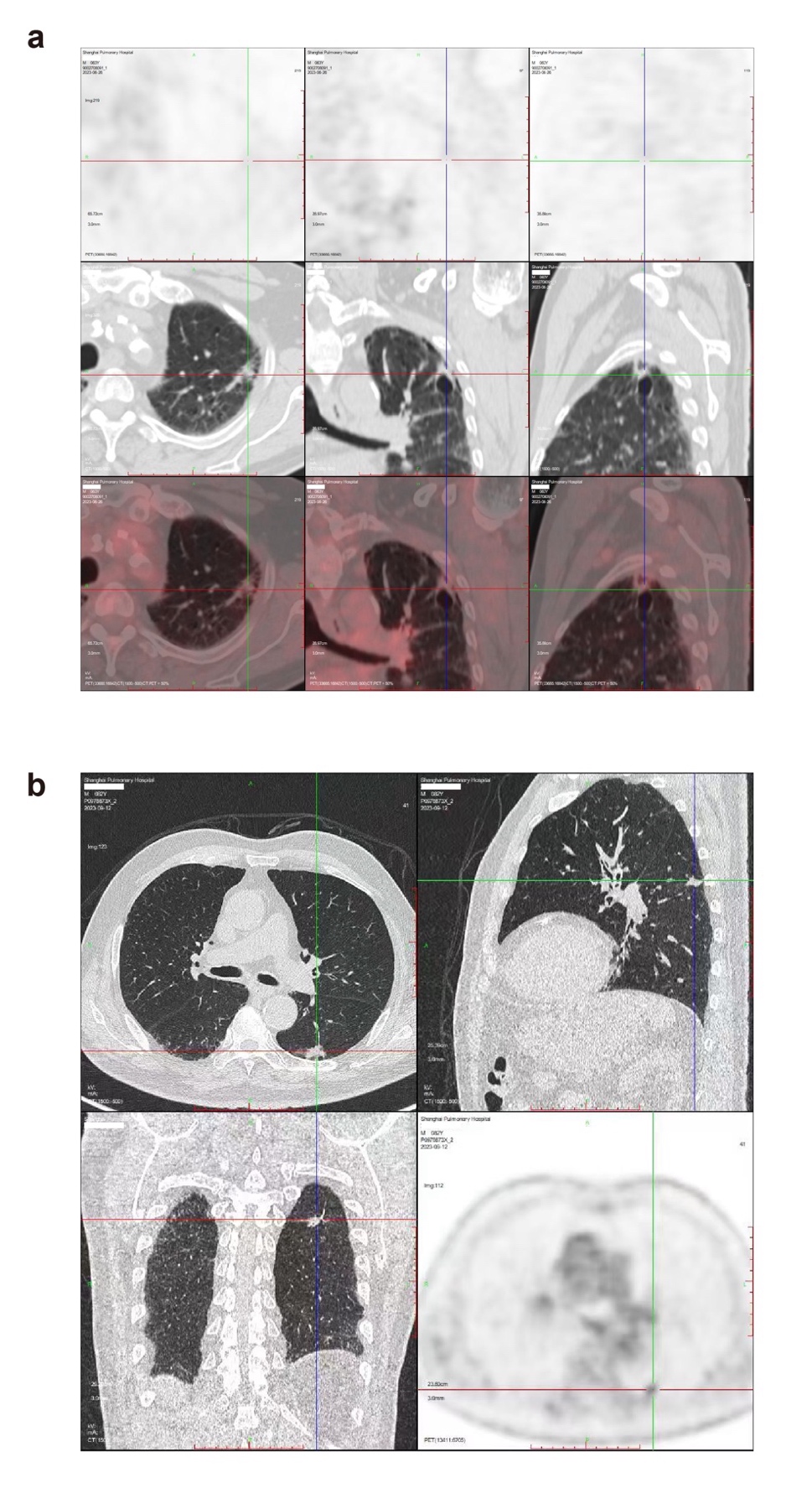


**Figure. S6.** Changes of cytokine levels pre and post (a) Thermal ablation (b) Cryoablation (c) Thermal ablation versus cryoablation. The Δconcentration (%) is calculated with a formula of (cytokine level post ablation-cytokine level pre ablation)/cytokine level pre ablation. Statistical analysis was performed using Student’s t-test. * represents p<0.05, ** represents p<0.01, *** represents p<0.001. The PET-CT images obtained before ablation from two patients with negative pathology.


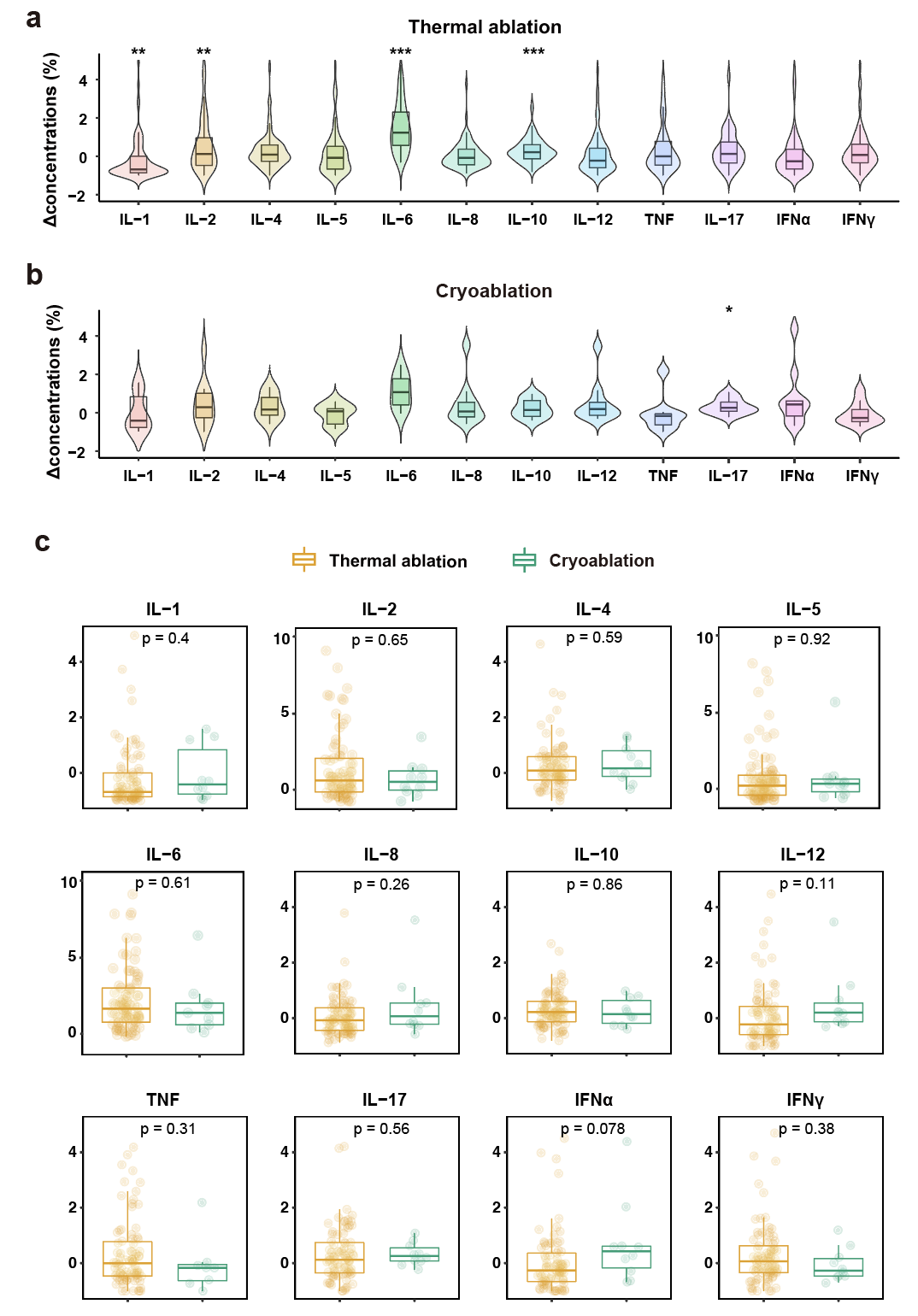


**Table S1.** Clinicopathological characteristics of patients received ablation to lung lesions with thermal or cryoablation

| **Characteristics** | **Total (n, %)** | **With cryo (n, %)** | **With thermal (n, %)** |
| --- | --- | --- | --- |
|  |  |  |  |
| **Gender** |  |  |  |
| male | 36 (85.7) | 13 (100.0) | 23 (79.3) |
| female | 6 (14.3) | 0 | 6 (20.7) |
| **Age (years)** |  |  |  |
| <65 | 22 (52.4) | 6 (46.2) | 16 (55.2) |
| ≥65 | 20 (47.6) | 7 (53.8) | 13 (44.8) |
| **ECOG-PS** |  |  |  |
| 0 | 14 (33.3) | 4 (30.8) | 10 (34.5) |
| 1 | 28 (66.7) | 9 (69.2) | 19 (65.5) |
| **Histology** |  |  |  |
| adenocarcinoma | 25 (59.5) | 9 (69.2) | 16 (55.2) |
| squamous carcinoma | 11 (26.2) | 3 (23.1) | 8 (27.6) |
| nsclc and other | 6 (14.3) | 1 (7.7) | 5 (17.2) |
| **PD-L1 expression** |  |  |  |
| unknown | 12 (28.6) | 3 (23.1) | 9 (31.0) |
| negative | 8 (19.0) | 1 (7.7) | 7 (24.1) |
| 1-50% | 12 (28.6) | 4 (30.8) | 8 (27.6) |
| ≥50% | 10 (23.8) | 5 (38.5) | 5 (17.2) |
| **Oncogenic mutation** |  |  |  |
| unknown | 12 (28.6) | 3 (23.1) | 9 (31.0) |
| wild type | 19 (45.2) | 8 (61.5) | 11 (37.9) |
| KRAS | 10 (23.8) | 2 (15.4) | 8 (27.6) |
| RET | 1 (2.4) | 0 | 1 (3.4) |
| **Disease extent** |  |  |  |
| intrathoracic | 26 (61.9) | 9 (69.2) | 17 (58.6) |
| extrathoracic | 16 (38.1) | 4 (30.8) | 12 (41.4) |
| **ICIs** |  |  |  |
| Camrelizumab | 32 (76.2) | 10 (76.9) | 22 (75.9) |
| Tislelizumab | 5 (11.9) | 3 (23.1) | 2 (6.9) |
| Pembrolizumab | 5 (11.9) | 0 | 5 (17.2) |
| **Total** | **42** | **13** | **29** |

Abbreviations: ECOG, Eastern Cooperative Oncology Group; PS, performance status; ICIs, immune checkpoint inhibitors.

**Table S2.** Detailed information of lung lesions received ablations between patients with thermal and cryoablations

| **Characteristics** | **Total (n, %)** | **With cryo (n, %)** | **With thermal (n, %)** |
| --- | --- | --- | --- |
|  |  |  |  |
| **Diameter of lung lesion,**  **(mean±SE)** | 2.174±1.050 | 1.608±0.556 | 2.428±1.125 |
| **Diameter of lung lesion** |  |  |  |
| ≤1cm | 7 (16.7) | 4 (30.8) | 3 (10.3) |
| ≤3cm | 26 (61.9) | 9 (69.2) | 17 (58.6) |
| >3cm | 9 (21.4) | 0 | 9 (31.0) |
| **Location of lung lesions** |  |  |  |
| left upper lobe | 8 (19.0) | 3 (23.1) | 5 (17.2) |
| left lower lobe | 8 (19.0) | 1 (7.7) | 7 (24.1) |
| right upper lobe | 16 (38.1) | 6 (46.2) | 10 (34.5) |
| right middle lobe | 3 (7.1) | 1 (7.7) | 2 (6.9) |
| right lower lobe | 7 (16.7) | 2 (15.4) | 5 (17.2) |
| **Pneumothorax or not,** |  |  |  |
| No | 27 (64.3) | 7 (53.8) | 20 (69.0) |
| Yes | 15 (35.7) | 6 (46.2) | 9 (31.0) |
| **Transthoracic drainage** |  |  |  |
| no drainage | 35 (83.3) | 12 (92.3) | 23 (79.3) |
| catheter | 6 (14.3) | 1 (7.7) | 5 (17.2) |
| chest tube | 1 (2.4) | 0 | 1 (3.4) |
| **Time to immunotherapy,**  **(mean±SE)** | 33.5±17.8 | 30.9±20.4 | 34.7±16.7 |
| **Total** | **42** | **13** | **29** |

**Table S3.** Treatment details of the three patients who withdrew consent

| **Patient** | **Group** | **Cycles of immunotherapy prior randomization** | **Time of immunotherapy prior randomization (months)** |
| --- | --- | --- | --- |
| **01005** | With ablation | 4 | 3.6 |
| **01024** | Without ablation | 6 | 5.2 |
| **01031** | With ablation | 7 | 6.1 |

**Table S4.** Clinicopathological characteristics of the immunogenic change exploring cohort

| **Characteristics** | **Total (n, %)** | **With cryo (n, %)** | **With thermal (n, %)** |
| --- | --- | --- | --- |
|  |  |  |  |
| **Gender** |  |  |  |
| male | 59 (60.8) | 8 (80.0) | 51 (58.6) |
| female | 38 (39.2) | 2 (20.0) | 36 (41.4) |
| **Age (years)** |  |  |  |
| <65 | 33 (34.0) | 1 (10.0) | 32 (36.8) |
| ≥65 | 64 (66.0) | 9 (90.0) | 55 (63.2) |
| **ECOG-PS** |  |  |  |
| ≤1 | 85 (87.6) | 8 (80.0) | 77 (85.5) |
| 2 | 12 (12.4) | 2 (20.0) | 10 (11.5) |
| **Histology** |  |  |  |
| adenocarcinoma | 71 (73.2) | 6 (60.0) | 65 (74.7) |
| squamous carcinoma | 13 (13.4) | 3 (30.0) | 10 (11.5) |
| nsclc and other | 13 (13.4) | 1 (10.0) | 12 (13.8) |
| **Smoking status** |  |  |  |
| ever | 42 (43.3) | 4 (40.0) | 38 (43.7) |
| never | 55 (56.7) | 6 (60.0) | 49 (56.3) |
| **Disease extent** |  |  |  |
| intrathoracic | 62 (63.9) | 7 (70.0) | 38 (43.7) |
| extrathoracic | 35 (36.1) | 3 (30.0) | 49 (56.3) |
| **Total** | **97** | **10** | **87** |
