# Supplementary material for: Ablation plus immunotherapy versus immunotherapy alone in patients of advanced NSCLC who develop oligo-residual disease after anti-PD-1/L1 therapy (BOOSTER): a randomized phase 2 trial
Source: Signal Transduct Target Ther. 2025 Nov 11;10:365. doi: 10.1038/s41392-025-02460-z (PMC12603194; doi:10.1038/s41392-025-02460-z)
Supplement: Supplementary file 2 — Study protocol [file 41392_2025_2460_MOESM2_ESM.pdf]

Ablation plus immunotherapy versus immunotherapy alone in patients of advanced NSCLC who develop oligo-residual disease after anti-PD-1/L1 therapy (Booster) : a randomized phase 2 trial

Study protocol

Trial ID number:	FKXRYJ-2020-02
Version number:	2.0
Version date:	April 26, 2020
Clinical trial leading unit:	Shanghai Pulmonary Hospital
Principal investigator:	Professor Shengxiang Ren

Contents

1 Study Background 5
1.1 Study Background and Significance 5
1.2 Summary of Ablation in Lung Cancer 8
2 Study purpose and endpoints 10
2.1 Study purpose 10
2.2 Study endpoints 10
3 Study design 11
4 Selection and withdrawal of subjects 11
4.1 Inclusion criteria 11
4.2 Exclusion criteria 12
4.3 Withdrawal from the study or termination of the study treatment 13
4.3.1 Withdrawal from the study 13
4.3.2 Termination of the study treatment 14
4.4 Early termination or suspension of the study 14
4.5 Definition of the end of the study 15
5 Study treatments 15
5.1 Regimens of systemic treatments 15
5.2 Dose adjustment of the study medication 16
6 Study procedures 16
6.1 Screening 16
6.2 Enrollment 18
6.3 Treatment period 18
6.4 End of Treatment (EOT) Visit 19
6.5 Unplanned visit 20
7 Evaluation 20

7.1 Efficacy evaluation 20
7.2 Safety evaluation 21
7.2.1 AEs 21
7.2.2 Laboratory safety evaluation 21
7.3 Biomarker exploratory analysis 21
8 Handling and reporting of AEs 22
8.1 AEs 22
8.1.1 Definition of AEs 22
8.1.2 Criteria for determining the severity of AEs 22
8.1.3 Determination of the relationships between AEs and study treatment 23
8.2 SAEs 23
8.2.1 Definition of SAEs 23
8.2.2 Hospitalization 24
8.2.3 Disease progression and death 24
8.2.4 Other antitumor treatments 25
8.2.5 SAE reporting system 25
8.3 AE/SAE collection and follow-up periods 25
9 Data analysis and statistical methods 26
9.1 Sample size calculation 26
9.2 Dropout cases 26
9.3 SAP 27
9.3.1 Efficacy end point 27
9.3.2 Safety analysis 27
9.3.3 Processing of missing data 28
10 Study management 28
10.1 Ethical norms 28
10.2 Informed consent 28
10.3 Study drug management 29

10.4 Protocol revision 29

10.5 Supervision 29

10.6 Quality Control and Assurance 29

10.7 Data management 30

10.7.1 Creating the EDC database 30

10.7.2 Data entry and verification 30

10.7.3 Data archiving 30

1 Study Background

1.1 Study Background and Significance

Lung cancer is the most prevalent and the leading cause of cancer death both worldwide and in China, with 1.8 million new cases and 1.6 million deaths annually globally, according to the World Health Organization (WHO) 。Surgical section is still the first choice for early-stage lung cancer, but only 20% -30% are suitable for surgical treatment. As the aging of the population, the proportion of middle-aged and elderly patients diagnosed as lung cancer is increasing. These patients often have comorbidities and are not suitable or able to tolerate conventional surgical resection. Minimally invasive treatments such as ablation has been increasing applied.

Minimal invasive ablation (MBI) of tumor is defined as the method of using extreme temperature to directly destroy tumor tissues and normal tissue 0.5 cm -1 cm around the target area in order to protect normal lung tissue under local anesthesia. Local ablation has multiple advantages, such as minimal invasion, high safety, conformal operation, less complications, high feasibility, quick recovery of patients, reliable effect, repeatable operation, and etc... Recently, it has become a reliable therapeutic method besides surgery, chemoradiotherapy, targeted therapy and immunotherapy. For early-stage lung cancer, local ablation has been widely applied. Ablation could achieve satisfactory local control rate and similar long-term survival compared to surgery and radiotherapy. For locally-advanced and advanced-stage lung cancer, local ablation could directly reduce tumor burden, thus facilitating the application of chemotherapy, immunotherapy, targeted therapy and radiotherapy. Since 2007-12, FDA has approved radiofrequency ablation for the treatment of pulmonary malignancies. Since 2009, NCCN guideline and CSCO guideline have also recommended ablation applied in patients with inoperable early-stage NSCLC or refusal to surgery.

Nowadays, more and more patients with advanced lung cancer have achieved residual

active lesions after systemic treatment. for these patients, local consolidative treatments including stereotactic radiotherapy and ablation therapy have been increasingly widely applied. Recently, it was demonstrated that local ablations to primary and metastatic sites of patients administrated with EGFR-TKIs at maximal response can significantly prolong overall survival (OS) than EGFR-TKI alone. Ablation to local residual lesions and progressive disease of targeted therapies have been demonstrated to augment the survival of patients with oncogenic mutations.

Local ablation mainly includes thermal ablation and cryoablation, including radiofrequency ablation (RFA), microwave ablation (MWA), laser ablation and high intensity focused ultrasound (HIFU). Thermal ablation causes irreversible coagulation and necrosis of tumor cells through local hyperthermia, thereby killing tumor。Local ablation also stimulates anti-tumor immune response, modulating tumor microenvironment (TME) thus have effects in immunomodulation。

Thermal ablation, including RFA and MWA, stimulates the release of tumor antigen, increasing Tumor necrosis factor (TNF)-alpha, Interleukin (IL) and Heat shock protein, thus to active host anti-tumor immune response。Series of basic and clinical studies have demonstrated that local ablation can induce the activation of tumor antigen-specific T lymphocyte and NK cells. In a murine urothelial carcinoma model, intratumoral Dendritic Cells (DCs) significantly increased after RFA, amplifying the T-cell immune response. Lin et al found that laser ablation can activate local and systemic Th1 type immune response in mouse model with liver metastasis of intestinal cancer. Den Brok et al found that the expression of costimulatory molecules such as CD80 and CD86 was upregulated after RFA, indicating that local tumor ablation can promote the presentation of antigen, migration and maturation of DCs。However, the anti-tumor immune response induced by local ablation is not enough to prevent recurrence and metastasis of tumor, which further indicates that ablation alone is not enough to induce a strong immune response. In addition, ablation has been found to stimulate an adaptive

T-cell immune response in sublethal regions, but this response is inhibited by changes in the tumor microenvironment, especially for incomplete ablation。

Although local ablation increases anti-tumor immune responses, but is not sufficient enough. Multiple studies have shown that local ablation only induces immune-enhancing responses at an early stage, but leads to lymphocyte depletion and immunosuppression later, especially in the case of incomplete ablation. Thus, the combination of local ablation with other treatment modalities are needed, especially with immune checkpoint inhibitors.

Immunotherapy with immune checkpoint inhibitors has progressed rapidly in NSCLC, which has been approved in first line and second line treatments of advanced NSCLC also in locally advanced NSCLC. Residual disease accounted for a significant proportion of patients received immunotherapy, in addition, residual diseases are generally the seeds of disease progression. Combinatory strategies including local consolidative treatment might prolong the survival benefit of immunotherapy.

In a study from the Yale Cancer Center, secondary resistance to immunotherapy in NSCLC was characterized by a predominance of progression of local lymph nodes, with oligo-progression as prevalent progression patten。Fifteen patients with acquired resistance to immunotherapy received locally treatments immediately (including stereotactic radiotherapy and local ablation) with discontinued systemic treatment, no severe adverse events were observed for local treatments. Median OS had not reached, while 2-year OS rate was 92%, with 11 patients resumed immunotherapy.

Another retrospective study evaluated the efficacy of immunotherapy of treatment beyond progression (TBP) in patients with advanced NSCLC treated with nivolumab or pembrolizumab。Patients were divided into two groups: 1. patients treated with "Classical" TBP, with continued immunotherapy during progression without the addition of other therapies;

2. patients with continued immunotherapy during progression with local treatments. It was showed that median PFS was 4.5 months, with median OS as 22.6 months, ORR as 26% and median DOR as 22.0 months. For those did not receive local treatments, BCR was significantly decreased than that of patients enrolled (31% vs. 58%, P=0.008). while, patients with local treatments showed increased BCR with marginal significance (92% vs. 58%, P=0.17).

Therefore, oligo residual lesions of immunotherapy may require intervention with local consolidate treatments. whether continued immunotherapy in combination with ablation can augment survival benefits is to be addressed.

1.2 Summary of Ablation in Lung Cancer

In recent years, thermal ablation techniques, represented by radiofrequency ablation (RFA) and microwave ablation (MWA), as well as cryoablation, have demonstrated definite efficacy in the treatment of pulmonary tumors. Currently, the most commonly used thermal ablation methods in clinical practice are radiofrequency ablation (RFA) and microwave ablation (MWA).

The working principle of RFA involves generating high-frequency electrical currents through electrodes within the tumor, causing rapid vibration and friction of electrons around the electrode needle, thereby producing heat to destroy tumor tissue. In contrast, MWA primarily relies on high-frequency electromagnetic waves to induce intense oscillations of charged ions and water molecules in the tissue, generating high temperatures. Compared to RFA, MWA has a higher heat production efficiency, allowing for the ablation of a larger tumor area in a shorter period. Additionally, it is less affected by the heat sink effect of blood flow, making it more suitable for treating tumors near blood vessels. Moreover, cryoablation (CRA) is a low-temperature ablation technique that has emerged as a novel treatment strategy in recent years. Cryoablation causes tumor tissue damage through repeated freeze-thaw cycles. Compared to thermal ablation, cryoablation can better preserve tumor antigens, thereby inducing a stronger cytotoxic T-cell response and enhancing the body’s anti-tumor immune response. A meta-analysis by Xu et al. of seven randomized controlled trials (RCTs) indicated that cryoablation significantly outperformed RFA in terms of three-year disease-free survival

rate and complication incidence.

Since 2009, the National Comprehensive Cancer Network (NCCN) clinical practice guidelines and the *Chinese Guidelines for Diagnosis and Treatment of Primary Lung Cancer* have recommended local thermal ablation for early-stage lung cancer patients who are unable to tolerate surgery. Cryoablation was also approved in 2018. Additionally, for patients with locally advanced late-stage non-small cell lung cancer (NSCLC), thermal ablation therapy can further reduce tumor burden, serving as part of a comprehensive treatment approach to create favorable conditions for subsequent systemic therapy. In the era of targeted therapy, for NSCLC patients with oligometastases or oligoprogression following EGFR-TKI treatment, combining local ablation therapy can further improve efficacy and prolong survival.

Our center previously conducted a study to evaluate the efficacy and safety of local ablation therapy in NSCLC patients with oligometastases undergoing first-line EGFR-TKI treatment. A total of 145 patients were included and divided into three groups: 51 patients received consolidative local ablation for all oligometastatic lesions (total LAT group), 55 patients received consolidative local ablation for only part of the primary tumor or oligometastatic lesions (partial LAT group), and 39 patients did not receive local ablation treatment (non-LAT group). The results showed that the median progression-free survival (PFS) was 20.6 months, 15.6 months, and 13.9 months in the total LAT, partial LAT, and non-LAT groups, respectively ($p<0.001$); the median overall survival (OS) was 40.9 months, 34.1 months, and 30.8 months, respectively ($p<0.001$). These findings indicate that local ablation therapy can improve survival benefits for lung cancer patients receiving first-line targeted therapy.

Additionally, Li et al. analyzed the efficacy of MWA in NSCLC patients with local progression after developing resistance to EGFR-TKI therapy. PFS1 was defined as the time from the initiation of targeted therapy to the first progression, while PFS2 was the time to the second progression after local treatment. The results showed that the median PFS1 was 9.5 months, while the median PFS2 was 8 months, with six-month and twelve-month PFS rates of 73.3% and 26.7%, respectively, demonstrating prolonged disease control.

Similarly, Ni et al. conducted a retrospective analysis of 54 NSCLC patients with EGFR

mutations who experienced central nervous system (CNS) oligoprogression following TKI treatment and subsequently received MWA. Among them, 28 patients continued with the same TKI after MWA (MWA group), while the remaining 26 patients underwent systemic chemotherapy after progression (chemotherapy group). The results showed no statistically significant difference in PFS1 between the two groups, but PFS2 was significantly longer in the MWA group compared to the chemotherapy group (8.8 months vs. 5.8 months, $p<0.01$). Multivariate analysis further confirmed that MWA was a favorable prognostic factor for PFS and OS. These findings suggest that, after targeted therapy resistance, performing thermal ablation for locally progressive lesions on top of the existing TKI treatment can further improve patient survival outcomes.

As cancer treatment enters the era of immunotherapy, the combination of local thermal ablation therapy with immunotherapy also offers new possibilities for further improving survival benefits in patients with advanced NSCLC.

2 Study purpose and endpoints

2.1 Study purpose

This study investigated the efficacy and safety of ablation combining continuous immunotherapy in NSCLC patients with oligo-residual disease (ORD) after anti-PD-1/L1 therapy.

2.2 Study endpoints

The primary endpoint is progression-free survival (PFS) since immunotherapy.

PFS since immunotherapy, defined as the time from immunotherapy initiation to Response Evaluation Criteria in Solid Tumours-defined progression or death

The secondary endpoints are as follows:

Overall survival (OS) since immunotherapy is defined as the duration from the initiation of immunotherapy until death from any cause. For subjects who have not experienced an OS event by the data collection cut-off date or who are lost to follow-up prior to the occurrence of an OS event, OS will be censored at the last known date of survival.

PFS since randomization is defined as the time from randomization to Response Evaluation Criteria in Solid Tumours-defined progression or death.

Overall survival (OS) since randomization is defined as the duration from the randomization until death from any cause. For subjects who have not experienced an OS event by the data collection cut-off date or who are lost to follow-up prior to the occurrence of an OS event, OS will be censored at the last known date of survival.

The incidence and severity of adverse events (AEs) and serious adverse events (SAEs) will also be monitored and recorded throughout the study.

3 Study design

The current clinical trial is a single-center, open-label, controlled, randomized phase II trial, aiming at investigating the efficacy and safety of local ablation with continued immunotherapy vs. immunotherapy alone in patients with oligo-residual state on immunotherapy. Patients met inclusion criteria but not exclusion criteria are to enrolled and randomized to either receiving local ablation with continued immunotherapy or immunotherapy alone.

In this study, the screening period is limited to a maximum of 28 days. Following the completion of screening assessments and evaluations, eligible participants will proceed to the treatment phase, with treatment cycles occurring every 28 days in accordance with the protocol. Biomarker detection will be conducted at baseline and after the first treatment cycle (4 weeks ± 3 days). For cycles 1 through 6 (5-28 weeks), tumor imaging will be performed every two cycles (8 weeks ± 3 days). Subsequently, tumor imaging will be conducted every three cycles (12 ± 3 days). Imaging assessments will be carried out in accordance with the RECIST 1.1 criteria. At the end of treatment or upon withdrawal from the study, subjects are required to visit the research center to undergo the relevant safety evaluations, imaging assessments, and biomarker detection. Additionally, subjects will return to the center 28 days after the last treatment for a final safety assessment.

4 Selection and withdrawal of subjects

4.1 Inclusion criteria

1 Patients those with age over 18 years old were cytologically/histologically diagnosed as advanced non-small cell lung cancer (NSCLC) (stage IIIB with multiple lobes involved/stage IV) and with Eastern Cooperative Oncology Group (ECOG)

Performance Status (PS) as 0-1.

2 Patients were administrated with PD-1/PD-L1 axis inhibitors (with/without chemotherapy) as first line treatments and achieved PR/SD after 4-6 cycles of immunotherapy and with number of residual lesions within three (maximum diameter of single lesion: 5 cm).

3 Patients were capable of receiving complete ablation to all of the residual lesions.

4 Adequate bone marrow function, defined as: a) Absolute neutrophil count (ANC) ≥1,500/mm³ (1.5 × 10^9/L) without granulocyte colony-stimulating factor support within 14 days. b) Platelet count (PLT) ≥75,000/mm³ (75× 10^9/L) without transfusion within 7 days. c) Hemoglobin (Hb) ≥9 g/dL (90 g/L) without transfusion within 7 days.

5 Adequate functions of heart, kidney and liver, defined as: a.) Left Ventricular Ejection Fraction >50% or within normal range. b) Creatinine clearance rate >45ml/min. c) Aspartate aminotransferase (AST) or alanine transaminase (ALT) <3×upper limits of normal (patients with liver metastases: <3×upper limits of normal). d) Total bilirubin <1.5 ×upper limits of normal (patients with Gilbert's syndrome: total bilirubin <4 ×upper limits of normal).

6 All patients who voluntarily agree to participate in the study, sign the informed consent form, demonstrate good compliance, and are willing to adhere to the follow-up plan.

4.2 Exclusion criteria

1 Patients with • Symptomatic brain metastases.

2 Patients with any of the following conditions prior to enrollment: myocardial infarction; severe or unstable angina; ventricular dysfunction (New York Heart Association [NYHA] grade 2 or above); persistent arrhythmias ≥ grade 2 (as per NCI CTCAE version 5.0); atrial fibrillation of any grade; coronary or peripheral artery bypass grafting; symptomatic congestive heart failure; cerebrovascular events (including transient ischemic attack); symptomatic pulmonary embolism.

3 Presence of a severe concomitant infection (e.g., requiring intravenous antibiotic, antifungal, or antiviral treatment as per clinical protocols) within 4 weeks prior to the

initiation of study treatment, or unexplained fever >38.5°C during the screening period or prior to the first treatment.

4 Presence of other serious persistent diseases or dysfunction of the organ system（those investigator regarding might impact or compromise the safety assessment）.

5 Women of childbearing age planning pregnancy or men of childbearing age; female patients with pregnancy or breastfeeding.

6 Patients could not comply with the instruction of the investigator/protocol.

7 Any other condition that, in the investigator's opinion, would make the subject unsuitable for participation in the study.

4.3 Withdrawal from the study or termination of the study treatment

4.3.1 Withdrawal from the study

Subjects may voluntarily withdraw from the trial at any time or may be required to withdraw by the investigator for reasons related to safety, behavioral concerns, or the inability to comply with the study's visit schedule or protocol requirements at the research center.

The reasons for withdrawal from the study may include, but are not limited to, the following:

- The subject withdraws informed consent to participate in the study and refuses further follow-up.
- The investigator determines that withdrawal is necessary, for example, if the subject loses the ability to make informed decisions due to incarceration or isolation.
- Loss to follow-up.
- The subject's death.
- The sponsor terminates the study.

All reasons for withdrawal must be documented in the case report form (CRF) and the subject's medical records.

It is important to note that "withdrawal of informed consent" refers to the subject's decision

to withdraw consent for further contact or for providing additional information to authorized personnel. Whenever possible, subjects should provide written notification to the investigator regarding their decision to discontinue participation and not to agree to further follow-up. Investigators should make every effort to explain the withdrawal of informed consent, including the subject’s decision to no longer receive the study drug or comply with the study visits as outlined in the protocol.

4.3.2 Termination of the study treatment

The termination of study treatment does not imply withdrawal from the study. Subjects who discontinue the study treatment are still required to complete the remaining study visits as stipulated by the protocol. The study treatment may be terminated under the following circumstances:

- The subject is requested to discontinue the study treatment.
- Medical imaging or clinical findings indicate disease progression.
- Due to the occurrence of clinical AEs, abnormal laboratory results, or other medical conditions, the subject is no longer deemed to benefit from continued study treatment.
- A general deterioration in the subject's health status renders them unable to continue participating in the trial.
- Significant deviations from the protocol are identified, such as the subject's ineligibility or noncompliance after enrollment.
- Loss to follow-up.
- The subject dies.
- Any other reasons, as determined by the investigator, that prevent the subject from continuing the study treatment.

4.4 Early termination or suspension of the study

If there are valid reasons, the study may be terminated or suspended prematurely. This may occur due to decisions made by the regulatory agency, changes in the ethics committee's opinion, concerns regarding the efficacy or safety of the study drug, or at the discretion of the principal

investigator (PI). The party responsible for the suspension or termination must provide written notice and document the reasons for the decision, which should be communicated to the investigator. The investigator is then required to promptly notify the ethics committee and provide the relevant justification.

4.5 Definition of the end of the study

The study is considered to have ended when the PI terminates the study following the achievement of the primary endpoint for the last enrolled subject, and the PI determines that a sufficient number of OS events have been collected.

5 Study treatments

5.1 Regimens of systemic treatments

In this study, subjects enrolled for analyses will be all administrated with immunotherapy with/without chemotherapy as first line treatment. Regimens used as immunotherapy are not limited, including but not limited to Pembrolizumab, Camrelizumab and Tislelizumab.

Pembrolizumab: 200mg once daily intravenously every 3 weeks.

Camrelizumab: 200mg once daily intravenously every 3 weeks.

Tislelizumab: 200mg once daily intravenously every 3 weeks.

Regimens used as chemotherapy are determined by the investigator per results of cytology and histology, including pemetrexed-based and paclitaxel-based doublet chemotherapy. For patients with inferior health status, single-agent chemotherapy is also permissible. Chemotherapy will be administrated for 4-6 cycles, and for those with adenocarcinoma maintenance of pemetrexed are preferred.

Subjects will continue treatment according to the study protocol until PD or intolerable toxicity occurs, or until the subject voluntarily discontinues treatment. Alternatively, treatment may be discontinued due to other medical events that, as determined by the physician, render the subject unsuitable for continued use of the combination therapy.

5.2 Dose adjustment of the study medication

Regimens of immunotherapy

When AEs related to immunotherapy occur, the investigators should adjust the treatment accordingly. AEs are to be graded as CTCAE 5.0. Discontinuation of immunotherapy will be conducted for AEs over grade 3. Once the AEs has resolved to grade 2 and below, re-administration may be considered, provided the AE does not pose significant safety risks. If the same AE recurs, the investigator may suspend or stop the immunotherapy permanently, or may require the subject to withdraw from the study. In all cases, the investigator must prioritize the safety of the subject. If the subject fails to recover within 4 weeks following treatment suspension due to toxicity, the subject should be withdrawn from the study in principle.

Chemotherapy Selected by the Investigator

When chemotherapy-related AEs occur, they should be managed according to the instructions of the selected drug and the investigator's clinical judgment. Any need for dose adjustments of chemotherapy should be based on the clinical situation, as determined by the investigator.

6 Study procedures

6.1 Screening

The screening period for the current study begins with the signing of the informed consent form and concludes with the initiation of ablation or the administration of immunotherapy or upon screening failure. Subjects in the ablation group must sign the informed consent form prior to undergoing the screening procedures outlined in this study. If laboratory examinations and imaging evaluations required for routine clinical care prior to informed consent fall within the specified time window and meet the study's criteria, the relevant data may be utilized.

Unless otherwise specified, the following screening procedures must be completed within 28 days before starting the study treatment:

- Written informed consent must be obtained from subjects of both groups.
- Demographic data such as sex, date of birth, height, and weight are collected.

- Tumor diagnosis details, including primary and recurrent lesions, the date of pathological diagnosis of metastatic lesions, pathological classification, pathological stage (TNM), genomic alterations, clinical stage, location of metastatic lesions, and best responses to systemic treatments.
- Previous disease and treatment history.
- Tumor imaging examinations: Ultrasound, computed tomography (CT) or magnetic resonance imaging (MRI) of the head, neck, chest, abdomen, and pelvis, along with bone scans, to assess tumor status. PET-CT are preferred.
- AEs: Documentation of AEs from the time of informed consent.
- Biomarker detection: Collection of 10 mL of blood (Streck tube x1) during the screening period for the detection of interleukin and cytokine levels.

The following screening procedures should be completed within 7 days prior to the initiation of study treatment:

- Eastern Cooperative Oncology Group (ECOG) performance status.
- Vital signs, including pulse, respiratory rate, temperature, and blood pressure.
- Comprehensive physical examination, including assessments of the general condition, head and face, skin, lymph nodes, eyes, ear, nose, and throat (ENT), oral cavity, respiratory system, cardiovascular system, abdomen, reproductive–urinary system, musculoskeletal system, nervous system, and mental status.
- Blood parameters, including red blood cell (RBC) count, Hb level, platelet (PLT) count, white blood cell (WBC) count, ANC, and lymphocyte count.
- Blood biochemistry, including alanine aminotransferase (ALT), aspartate aminotransferase (AST), gamma-glutamyl transferase (y-GT), total bilirubin (TBIL), direct bilirubin (DBIL), alkaline phosphatase (AKP), blood urea nitrogen (BUN) or urea (preferably BUN), total protein (TP), albumin (ALB), creatinine (Cr), blood glucose (GLU), potassium (K+), sodium (Na+), calcium (Ca2+), magnesium (Mg2+), and chloride (Cl-).
- Coagulation function, including activated partial thromboplastin time (APTT),

prothrombin time (PT), thrombin time (TT), fibrinogen (FIB), and international normalized ratio (INR).

- Urine routine test, including WBCs, RBCs, and urine protein. If the urine protein level is ≥2+, a 24-hour urine quantitative protein measurement should be performed.
- 12-lead electrocardiogram (ECG).
- Echocardiography, including evaluation of left ventricular ejection fraction (LVEF).

6.2 Enrollment

- The inclusion and exclusion criteria are applied to screen both the patients in both groups.
- For laboratory tests before randomization (routine blood tests, routine urine tests, blood biochemistry tests, coagulation function tests, hormone status tests and thyroid function tests) and ECG tests, if the corresponding baseline laboratory tests are performed within 7 days before, there is no need to retake the tests.
- The subjects received treatment.

6.3 Treatment period

The treatment period begins once the randomization is finished. The randomization should be administered as soon as possible after the screening assessments have been completed and the inclusion and exclusion criteria have been confirmed and after the collection of written consents. For patients randomized to ablation group, ablation is to be conducted within 3 days post randomization. Re-administration of immunotherapy is to be determined by the investigator once the subjects recover from the treatment of ablation with CT scans showing no signs of pneumonia. For patients randomized to control group, immunotherapy is to be conducted within 3 days post randomization with treatment cycle defined as 21 days. All assessments and evaluations scheduled during the treatment period (excluding imaging examinations) must be completed within 3 days prior to the randomization. For the ECOG performance status, if the relevant examination is conducted within 7 days before randomization, it does not need to be repeated.

The following evaluations should be completed before the administration of immunotherapy of each cycle (±3 days):

- ECOG score
- Vital signs
- Comprehensive physical examination
- Routine blood tests
- Blood biochemistry
- Urine routine test
- 12-lead ECG
- AE records
- Records of the combined medications and concomitant treatments.

Evaluations that are not required for every cycle during the treatment period:

- Tumor imaging examinations: Ultrasound, CT or MRI of the chest and abdomen will be performed (for patients with baseline brain or pelvic metastases, CT/MRI of the corresponding regions should also be performed). The conditions for the imaging examinations should match those used at baseline (including layer thickness, contrast agents, etc.). For subjects without baseline bone lesions, routine bone scans are generally not performed unless clinically indicated. For subjects with baseline bone lesions, bone scans are recommended every 6 cycles (24 weeks). For subjects without baseline CNS metastases, routine MRI are generally not performed unless clinically indicated. For subjects with baseline CNS metastases, MRI are recommended every 6 cycles (24 weeks). The allowed time window for tumor imaging examinations is ±7 days. The investigator may order additional imaging assessments as needed, based on the subject's condition.

6.4 End of Treatment (EOT) Visit

The current study does not include a requirement for an End of Treatment (EOT) visit.

6.5 Unplanned visit

If the subjects require unplanned follow-up due to the occurrence of AEs during the trial, the following items should be recorded:

- Records of the combined medication/concomitant treatment
- AE records
- Records of the relevant examinations performed (including imaging examinations, if any).

7 Evaluation

7.1 Efficacy evaluation

Primary endpoint:

Progression free survival (PFS)：time interval from randomization or the initiation of immunotherapy to disease progression (target lesion included) or patient’s death.

Secondary endpoint：

Overall survival (OS)： time interval from randomization or the initiation of immunotherapy to patient’s death (death related to cancer or other disease).

Other secondary endpoint：

Time to local progression (TTLP): time interval from local ablation to progression of target lesions, only applied for patients receiving ablations.

Efficacy evaluation of ablation：

According to the Chinese experts' consensus on ablations to primary/metastatic lung cancer, ablations shall be classified as complete ablation and incomplete ablation. Complete ablation indicates tumor disappearance, cavity formation, focal fibrosis or fibrous scar formation, shrinkage of solid nodules, or no change or growth of solid nodules but with no enhancement in CT with contrast or no up-take in 18F-FDG PET/CT; pulmonary atelectasis or lesions in pulmonary atelectasis but with no enhancement in CT with contrast or no up-take in 18F-FDG PET/CT. Incomplete ablation indicates incomplete cavity formation with partially solid nodule with enhancement in CT with contrast or up-take in 18F-FDG PET/CT；incomplete focal fibrosis with partially solid nodule with enhancement in CT with contrast or up-take in 18F-FDG PET/CT; no change or growth of solid nodule with enhancement in CT with contrast

or up-take in 18F-FDG PET/CT.

7.2 Safety evaluation

7.2.1 AEs

The evaluation of AEs includes the incidence per patient, severity (graded according to the NCI CTCAE version 5.0), start and end dates, whether the event is classified as a SAE, its relationship to the study treatment, and the outcome.

AEs occurring during the study, including any signs, symptoms, and treatments related to the AE, will be recorded on the AE page of the CRF. The signs and symptoms of AEs occurring during the screening period will be documented as comorbidities on the past medical history page of the CRF.

Adverse events related to ablation are also categorized and assessed with SIR system, mild AEs defined as no therapy or nominal therapy; moderate AEs defined as needs for treatment, short hospital stay (< 48 h) , need for intensive treatment, unplanned medical care, hospital stay significantly prolonged (>48 h), persistent adverse effects, and death. Adverse reactions include pain, post-ablation syndrome, cough and pleural reaction.

7.2.2 Laboratory safety evaluation

Blood samples for hematology and blood biochemical tests will be collected according to the specified testing procedures. Analyses of these samples will be performed at the laboratories of the hospitals participating in the study.

7.3 Biomarker exploratory analysis

Blood samples (10 mL in 1 tube) of patients received with ablation will be collected from enrolled patients prior and post ablation (within 48 hours) for the assessment of exploratory biomarkers of interleukins, cytokines and immune cell subsets. Instructions for handling, storing, and transporting these samples will be provided in the research laboratory handbook.

Interleukin levels and cytokine levels are to be assessed using flowcytometry via BD Cytometric Bead Array (CBA) in the Clinical Laboratory Department of Shanghai Pulmonary

Hospital.

8 Handling and reporting of AEs

8.1 AEs

8.1.1 Definition of AEs

An AE is any unfavorable medical occurrence in a subject who has received medical treatments, and it does not necessarily need to have a causal relationship with the treatment. An AE may include any unintended or unfavorable sign (such as an abnormal laboratory finding), symptom, or disease temporally associated with the application of medical treatments, regardless of whether it is considered related to medical treatments.

It is the responsibility of the investigators, based on their knowledge and expertise, to determine which circumstances or abnormal laboratory findings should be classified as AEs.

The investigators must document all AEs and SAEs in detail, including the term and description of the relevant symptoms, the time of occurrence, severity, relationship to the medical treatments, duration, actions taken to resolve AEs, as well as the outcomes and results.

8.1.2 Criteria for determining the severity of AEs

The NCI-CTC AE version 5.0 is used to grade.

Table 1 Criteria for determining the severity of AEs

Grade	Clinical description of severity
1	Mild; asymptomatic or with mild symptoms; clinical or diagnostic observations only, with no intervention required.
2	Moderate; minimal, local, or non-invasive intervention required; limits age-appropriate instrumental activities of daily living (ADL).
3	Severe or medically significant, but not immediately life-threatening; hospitalization or prolongation of hospitalization required; disabling; limits self-care ADL.

4	Life-threatening consequences; urgent intervention required.
5	Death related to the AE.

8.1.3 Determination of the relationships between AEs and study treatment

The collection of data on AEs begins with the signing of the informed consent form, regardless of whether the events are related to the treatment or the use of any other treatment. During the treatment period, any adverse reactions or abnormal changes in objective laboratory examination indicators, along with the severity, duration, treatment measures, and outcomes of the AEs, should be accurately documented. Investigators must thoroughly assess and determine whether the AE is related to the study treatment.

8.2 SAEs

8.2.1 Definition of SAEs

SAEs are defined as medical occurrences that result in hospitalization or prolonged hospitalization, disability, impairment of the ability of working, life-threatening conditions, death, or congenital malformations, occurring during a clinical trial. These events include the following:

- Events leading to death;
- Life-threatening events (defined as situations where the subject is at immediate risk of death at the time of the event);
- Events requiring hospitalization or prolonged hospitalization;
- Events leading to permanent or severe disability, incomplete function, or impairment of ability of working.

Additionally, other important medical events are those that, while not immediately life-threatening and not resulting in death or hospitalization, may, according to reasonable medical and scientific judgment, cause harm to the subject or necessitate intervention (such as medication or surgery) to prevent serious consequences as defined above.

8.2.2 Hospitalization

In clinical studies, any AEs that result in hospitalization (even if for less than 24 hours) or prolonged hospitalization should be considered SAEs. However, the following situations do not constitute hospitalization under the SAE definition:

- Admission to rehabilitation institutions;
- Admission to nursing homes;
- Routine emergency room visits (<24 hours);
- Same-day surgeries (e.g., outpatient, same-day, or ambulatory surgeries);
- Hospitalization for social reasons (e.g., medical insurance reimbursement);
- Hospitalization or prolonged hospitalization unrelated to the worsening of AEs. For example, hospitalization due to preexisting conditions, without new AEs or worsening of existing conditions (e.g., persistent laboratory test abnormalities from before the study);
- Hospitalization for management purposes (e.g., annual routine physical examination);
- Hospitalization as required by the study protocol during the clinical trial (e.g., per the trial's procedural requirements);
- Elective hospitalization (e.g., for elective surgery) not related to the worsening of an AE;
- Scheduled treatments or surgical procedures as specified in the trial protocol or the subject’s individual baseline information;
- Hospitalization solely for the use of blood products.

8.2.3 Disease progression and death

In the study population of this trial, "disease progression" is considered an expected outcome and should not be reported as an AE. However, when disease progression occurs, any events associated with it should be reported as AEs.

The term "death" should not be classified as an AE or SAE; rather, it should be regarded as the outcome of the event. The event that causes or leads to death should be recorded as an

AE or SAE. If the cause of death is unknown and cannot be determined at the time of reporting, the AE or SAE should be recorded as "death from unknown causes."

8.2.4 Other antitumor treatments

SAEs are recorded from the time the subjects signed the informed consent form to 28 days after the last application of medical treatments. If the subjects start receiving other antitumor treatments within 28 days, for non-death SAEs, unless they are suspected to be related to the study drugs, the reporting period ends at the start of new antitumor treatment. If death occurs within 28 days after the last application of medical treatments, regardless of whether the patient receives other treatments, it is reported as an SAE.

8.2.5 SAE reporting system

The collection period of SAEs should be from the time when the subjects sign the informed consent form to the 28 days (including Day 28) after the last application of medical treatments. In an SAE, whether it is the first report or the follow-up report, the investigator must immediately fill in the *Severe Adverse Event Reporting Form*, sign and date it, and report it to the relevant units in a timely manner in accordance with regulatory requirements. SAEs, including symptoms, severity, correlation with the study drugs, time of occurrence, treatment time, measures adopted, time and methods of follow-up, and outcomes, should be recorded in detail. If the investigator believes that the previously reported SAE is misreported, the investigator can correct, withdraw or downgrade the SAE in the follow-up report and report it according to the SAE reporting procedure.

8.3 AE/SAE collection and follow-up periods

AE/SAE information is collected from the time when the subjects sign the informed consent form until the end of the safety follow-up period, that is, 28 days after the last application of medical treatments (whichever is longer) or screening failure.

At each visit, the investigators should ask about the AEs/SAEs that occur since previous visit. In addition, follow-up information is provided in a timely manner according to the queries received.

All AEs/SAEs should be followed up until the resolution of symptoms and the clinically relevant changes in laboratory values return to the baseline and/or ≤ Grade 1, with a reasonable explanation (such as loss to follow-up, death) or the event being ultimately confirmed to be unrelated to the study drug or the study processes at the end of the safety follow-up period.

9 Data analysis and statistical methods

The detailed summary and statistical analysis methods for the data collected in this study will be included in a statistical analysis plan, which will be finalized and filed by the PI. If any change to the study protocol, which has a significant effect on the SAP determined by the PI, the SAP will need to be revised simultaneously to be consistent with the study protocol.

9.1 Sample size calculation

The primary objective of this study is to preliminarily evaluate t the efficacy and safety of ablation combining continuous immunotherapy in NSCLC patients with oligo-residual disease (ORD) after anti-PD-1/L1 therapy. The primary endpoint of the study is the progression-free survival (PFS) since immunotherapy.

The sample size was calculated with the 2-sided significance level of 0.05 and 80% statistical power using a 2-sample log-rank test. We presumed a lengthened PFS of 6 months to be observed in cohort of Arm A with estimated PFS of Arm B as of 6 months since randomization, which translated to a hazard ratio (HR) of 0.5. Estimates for PFS were based on data from randomized phase III trials, meta-analyses and retrospective studies. With randomization ratio as 2:1 and expulsion rate of 10%, a total sample size of 137 patients with 91 patients for Arm A and 46 for Arm B was estimated to achieve the desired statistical power.

9.2 Dropout cases

All the subjects who sign the informed consent form and are qualified to enter the trial have the right to withdraw from the clinical trial at any time. Regardless of when and for what

reason, as long as the subjects do not complete at least one dose of the trial drug and are unable to undergo the safety and efficacy evaluation, they are considered drop-out cases (subjects who experience disease progression and have clear medical evidence after enrollment are not considered drop-out cases). When the subject drops out, the investigator must fill in the reasons for the drop-out in the CRF, complete the evaluation items that can be completed, and carefully fill in the record of the last visit in the CRF. The number of dropouts due to AEs, which are ultimately determined to be related to the study drug after follow-up, should be recorded in the CRF, and the investigator should be informed. The subjects who withdraw from the study after screening but do not obtain the ID number are not considered drop-out cases.

Subjects who withdraw from the study cannot re-enter the study, and their data cannot be used again.

9.3 SAP

9.3.1 Efficacy end point

Chi-square test or Fisher's exact test was used to compare the clinical characteristics. Median PFS and OS were estimated by Kaplan-Meier method and compared by Log-rank test. Cox proportional hazard model was performed to calculate the hazard ratio (HR) and 95% confidence interval (CI) to determine the survival differences in subgroup analysis. Student's t-test and Wilcoxon rank-sum test was utilized to compare the change of interleukin levels. All p values are based on a two-sided hypothesis with exact calculations, a value of $p<0.05$ was considered statistically significant. All of the statistical analyses were performed using SPSS version 22.0 Software (SPSS, Inc, Chicago, IL).

At the same time, the number and percentage of cases and the number and percentage of censored cases of OS/PFS events are calculated, and they are classified according to the cause of censoring.

9.3.2 Safety analysis

Safety analysis will be conducted on SS, descriptive statistics are summarized on the following data (including but not limited to): study termination by the subjects; suspension

or dose reduction of the study treatment, AEs.

9.3.3 Processing of missing data

The management rule of missing data will be described in detail in SAP as needed.

10 Study management

10.1 Ethical norms

The clinical trial must comply with the *Declaration of Helsinki* (2008 edition), Good Clinical Practice (GCP) promulgated by the NMAP (formerly the CFDA), and relevant laws and regulations. Before starting the study, approval from the Ethics Committee of the responsible institution must be obtained. During the clinical study, certain modifications to the trial protocol should be reported to the ethics committee and recorded. The investigator is responsible for regularly submitting an interim report according to the relevant requirements of the ethics committee, and after the end of the trial, the investigator should notify the ethics committee that the trial is completed.

10.2 Informed consent

Before receiving drug treatment in the trial, the subjects in the experimental group provide informed consent for participation in the trial to protect the legitimate rights and interests of the subjects. The investigators are responsible for completely and comprehensively introducing the purpose of the study, the effects of the drugs, the possible toxic and side effects and the possible risks to the subjects or their designated representatives and should let the subjects know their rights, the risks to be assumed, and the benefits. Conversation is a very important process of informed consent. If the subject and his legal representative are illiterate, a witness should participate in the informed consent process. After the subject or his legal representative gives verbal consent, the subject and witness sign the informed consent form. The signatures of the witness and the subject should be obtained on the same day. The informed consent form should indicate the version number and date.

10.3 Study drug management

The management, distribution and recall of the clinical medications used in this trial are the responsibility of assigned personnel. The investigators must ensure that all study drugs are used only for the subjects participating in the clinical trial and the dosage and usage should be in accordance with the trial protocol. The remaining drugs will be returned to the sponsor. Clinical study drugs should not be transferred to any nonclinical trial participant.

10.4 Protocol revision

Except for the investigator, no one can make changes to the protocol. Any necessary changes to the protocol should be made in the form of protocol amendments and should be submitted to the ethics committee for approval or the record after the PI agrees and signs, and the details of the previous modifications should be stated in the protocol.

10.5 Supervision

The major research unit appoints personnel with appropriate medical, pharmacy, or related professional degrees, the necessary training, and the familiarity with the GCP and relevant laws and regulations as the supervisors of the clinical trial to monitor and report on the progress of the trial, verify the data, protect the rights and interests of the subjects in the clinical trial, ensure that the data recorded and reported in the trial are accurate and complete, and ensure that the trial follows the approved protocols, the GCP and relevant laws and regulations.

The monitoring of AEs and SAEs in clinical trials ensures that all AEs are accurately, reliably, and timely recorded and reported.

10.6 Quality Control and Assurance

- Investigators must be physicians trained in clinical trials and work under the guidance of senior professionals.
- Pretrial inspection of the clinical ward must meet the standardized requirements to ensure that first aid is well-equipped

- Professional nursing staff will administer medications to the subjects and understand drug usage in detail to ensure compliance.

- Each research center must strictly follow the study protocol and truthfully fill in the eCRF

- The supervisors should follow the standard operating procedures, supervise the execution of the clinical trial, confirm that the recorded and reported data are correct and complete and all eCRFs are correctly filled in and are consistent with the original data, in order to ensure that the trial is implemented in accordance with the clinical study protocol.

- Once an SAE occurs, each research center must report it in accordance with the GCP.

10.7 Data management

10.7.1 Creating the EDC database

The data administrator establishes the research data acquisition system and database according to the study protocol and provides them for online use before the subjects are enrolled. Before use, all EDC users should be adequately trained.

10.7.2 Data entry and verification

After the investigator fills in and submits the eCRF, the supervisor, data administrator, and medical personnel should review the relevant data one by one, and the investigators should be asked to answer the problems found during the review. After the data verification is completed, the investigator should sign the eCRF.

10.7.3 Data archiving

After the completion of the study, the EDC system will generate the subject's eCRF in PDF format and save it on a nonrewritable compact disc (DVD), which will be submitted to the sponsor and each institution for future audit.
